# An integrated evolution-aware meta-learning framework with adversarial morphological augmentation for zero-day threat detections

**DOI:** 10.3389/frai.2026.1814012

**Published:** 2026-06-02

**Authors:** Kavitha Lanka, Kareemulla Shaik

**Affiliations:** School of Computer Science Engineering, VIT-A.P University, Amaravati, India

**Keywords:** adversarial data augmentation, analysis, evolutionary threat modeling, meta-learning, uncertainty-aware detection, zero-day attacks

## Abstract

**Introduction:**

Most modern threat detection frameworks rely on fixed class definitions and retrospective signatures derived from historical data, limiting their ability to adapt to evolving attack behaviors. However, contemporary threats are increasingly polymorphic, adaptive, and include a growing number of zero-day attacks, making traditional pattern-matching approaches insufficient. With expanding attack surfaces across cloud environments, IoT systems, and heterogeneous infrastructures, detection systems must move beyond static similarity matching and instead account for the evolutionary nature of threats. Despite advances, both deep learning-based and traditional anomaly detection approaches face critical limitations. Deep learning models often suffer from data scarcity, concept drift, poor uncertainty calibration, and limited causal interpretability, while conventional anomaly detection methods exhibit slow training, weak generalization, and reduced adaptability to novel attack patterns.

**Methods:**

To address these challenges, we propose M^3^-GAZE (Meta-Morphological GAN-Augmented Zero-day Detection Engine), which formulates threat detection as an evolutionary inference problem. The framework consists of five analytically distinct yet interdependent components. First, the Latent Morphology Spectrum Extraction (LMSE) module learns a continuous latent representation capturing structural invariants of threats across morphological variations, enabling a more flexible representation than discrete labels. This latent space is utilized by the Adversarial Evolutionary GAN (AE-GAN) to generate evolution-consistent synthetic samples that reflect unseen attack variations while minimizing unrealistic artifacts. These synthetic samples enhance the training of the Meta-Adaptation Framework for Threat Intelligence (MAFTI), which learns adaptation strategies across multiple evolutionary tasks, enabling accurate zero-day detection under limited data conditions. The enriched latent space further supports Adversarial Uncertainty Calibration Layer (AUCL), which evaluates epistemic uncertainty by introducing controlled adversarial perturbations. This transforms uncertainty into an action able early warning signal. Finally, the Causal Evolutionary Threat Graph Synthesizer (CETGS) constructs a temporal causal graph that explains threat evolution, detection decisions, and propagation dynamics.

**Results and discussion:**

Overall, M^3^-GAZE improves zero-day recall, enhances few-shot adaptability, calibrates uncertainty, reduces false negatives, and provides interpretable, temporally aware threat detection.

## 1 Introduction

There have been shifts in the malware families and attack signatures that pose a threat to cyberspace. Polymorphism, behavior obfuscation, and rapid mutation cycles are several techniques that attackers employ to avoid being discovered (Ramalingam et al., 2025; Paya and Gómez, 2025; Sable et al., 2025). Because of the rapid expansion of cloud-native platforms, ecosystems for the Internet of Things, and software-defined networks, it is becoming increasingly expensive to identify threats that are not identified immediately or that are unstable (Saurabh et al., 2024; Almuflih et al., 2024; Ali and Chen, 2025). Traditional intrusion detection and signature-based methods are ineffective in situations where there is a great deal of uncertainty, a limited amount of data that has been tagged, and attacks that alter the meaning of what they signify.

The capabilities of machine learning and deep brain design make it simpler to identify attack patterns; nevertheless, these capabilities are only useful after the event. Several techniques are ineffective against zero-day assaults and adversarial strategies because they make use of closed-world classification, predetermined feature distributions, or many labeled samples. These techniques are among the many that are available. Static latent spaces, which do not have any structure continuity between attack variations in process, are utilized by a significant number of techniques for the purpose of developing deep representation learning and discovering anomalies. Therefore, even little modifications that are nonetheless significant from a semantic standpoint can result in detection that is less accurate, which can lead to false negatives and mistrust among analysts.

The purpose of Generative Adversarial Networks, often known as GANs, is to fill in gaps in the data by creating negative instances. Currently, the way augmentations are performed frequently alters the appearance of individuals for no apparent reason, which results in an increase in noise rather than diversity. Although few-shot and meta-learning can undergo rapid transformations, the majority of these techniques are more concerned with task-level generalization across preset classes than they are with altering the way assaults are carried out. Furthermore, an estimation of the degree of uncertainty is not included, which results in confidence scores that are not calibrated properly and conceal early warning indications of operational issues. According to the findings of this research, the limitations of existing systems are not a result of model capabilities but rather of representation theory. Each attack is a one-time occurrence; there is no discernible pattern for them. It is not enough for threat detection to rely solely on static classification; it must also make use of evolutionary inference to investigate structural morphology, mutation histories, and the movement of causes throughout time. For this shift to take place, we require an integrated system that is capable of learning invariant representations, providing data that are consistent with evolution, adapting rapidly to scenarios that only require a few shots, and communicating uncertainty in a manner that is both principled and easy to comprehend. A Meta-Morphological GAN-Augmented Zero-day Detection Engine, which is referred to as M3-GAZE, is presented in this study. It views the detection of threats as a challenge that requires evolutionary learning. The pipeline combines adversarial evolutionary data creation, meta-adaptive learning, uncertainty calibration, and causal graph synthesis to produce cyber defense that is adaptable, explainable, and robust in situations where the attacker is subject to change for real-time scenarios.

### 1.1 Motivation and contributions

The main reason behind the research is based on the fact that there are significant gaps between the number of cyberattacks that occur and the ability of detection technology to recognize those attacks. Cyberattacks tend to evolve by increments and therefore can be categorized into three types of attack mechanisms. These include structural changes or modifications to existing attacks; variant attacks at the execution level (i.e., different ways an attack may execute); and context-based attacks which may use variables that exist within the operating environment. Current detection approaches using machine learning (including deep learning) do not account for evolutionary relationships between attacks resulting in poor decision boundaries (i.e., brittle decision boundaries).

Moreover, they also result in delayed zero-day recognition capabilities. Another problem facing detection researchers is that false confidence has become a major issue in large-scale security operations. That is, due to the lack of understanding regarding how well a particular model represents the world, models have been developed that provide certainty about their decisions when in reality they are misinformed. Based upon these issues, a new approach to detection that accounts for evolutionary behavior in detected entities, provides responses to data scarcity, and uses uncertainty as an actionable output signal instead of treating uncertainty as an after-thought statistically is necessary.

This motivation allows the authors of the study to identify several contributions to the field. First, the authors introduce Latent Morphology Spectrum Extraction (LMSE)—a new way to represent attacks such that each attack is represented in a continuous evolutionary space where structural properties are preserved across all forms of polymorphism. Second, the authors develop a synthetic attack generator called Adversarial Evolutionary GAN (AE-GAN) whose goal is to generate attacks that reflect evolving mutations to expand threat coverage under realistic semantic constraints. Third, the authors propose a new few-shot threat detector called Meta-Adaptive Few-Shot Threat Inducer (MAFTI) that learns adaptive dynamics to detect zero-day threats with very little training data. Fourth, the authors develop a layer for validating uncertainty estimates called Adversarial Uncertainty Calibration Layer (AUCL) whose purpose is to provide early warning signals and calibrated confidence levels across changing distributions. Fifth, the authors develop a causal graph synthesizer called Causal Evolutionary Threat Graph Synthesizer (CETGS) whose function is to synthesize causal graphs representing how assaults progress through time along with why the attacker chose one path vs. another given the detection outputs. As a whole, M3-GAZE presents a single detection methodology that considers evolutionary aspects of detected entities that far exceeds static classification methods and reactive defense mechanisms. Therefore, M3-GAZE provides improved detection accuracy, robustness, interpretability, adaptability, and operational trust that make it suitable for future generations of zero-day threat detection systems.

## 2 Review of existing models used for analysis

Studies that have recently been conducted about discovering zero-day threats, flexible cybersecurity, and intelligent threat intelligence have revealed that although concepts are expanding, structures are getting more disconnected. The initial efforts demonstrate a shift away from systems that are driven by signatures and toward systems that are driven by learning and intelligence. ZeSAI is a program that was developed by Ramalingam et al. It is designed to detect email viruses by utilizing zero-shot learning, hybrid networks, and threat intelligence capabilities (Ramalingam et al., 2025). It is of greater significance to make use of semantic reasoning in situations where labels are not given. When it comes to software-defined perimeters and identity-centric zero-trust systems (Paya and Gómez, 2025), Paya and Gómez emphasize that the context of access is the determining factor in how effectively threats are discovered. Sable et al. employ bioinspired incremental learning in cloud-native environments to identify zero-day threats that are constantly developing (Sable et al., 2025).

The research conducted by Saurabh et al. demonstrates that static models are unable to prevent coordinated behaviors in HMS IDS. To accomplish this, they combine threat information streams with sets of zero-day vulnerabilities specific to the Internet of Things (IoT) and APT detection logic (Saurabh et al., 2024; Almuflih et al., 2024). The work that Almuflih and others have done on the detection of Internet of Things attacks takes into account zero-day modifications and restricted resources, all while optimizing and paying attention to the process. As the quality of writing improves, architecture begins to rise to the forefront. The neuromorphic quantum adversarial learning for encrypted DNS traffic that Ali and Chen have developed provides a signal that in the future, computers that are based on biology may be able to defeat adversarial stealth (Ali and Chen, 2025) sets.

Initially, as per Table 1, the detection of malware on Android is accomplished by Ransom Hunter through the utilization of graph convolutional networks and code embeddings with structure semantics (Ebrahimifard et al., 2025). Mohamed et al. demonstrate how adaptive WavePCA-Autoencoders and hybrid feature compression can be utilized to locate vulnerabilities in systems that have larger attack regions (Mohamed et al., 2025). When it comes to Internet of Things zero-day detection, attention fusion models are aware of the requirement for alerts that can be comprehended (Krishnan et al., 2025). The findings of research that simultaneously examine the Android botnet and the cloud trust architecture demonstrate that each platform possesses its own set of real-time boundaries and areas of expertise (Seraj et al., 2024; Lilhore et al., 2025). Teamwork, adaptation, and the integration of systems are the topics that are discussed in the subsequent articles in a series. Distributed intelligence that can adapt to scale and heterogeneity is demonstrated by mobile edge network multi-agent explainable reinforcement learning (Saeed et al., 2025). It is not possible to use a single monitor in layered artificial intelligence, zero trust, or threat intelligence (Khule et al., 2025).

**Table 1:** Model's integrated review analysis.

References	Method	Main objectives	Key findings	Limitations
Ramalingam et al. (2025)	ZeSAI (zero-shot hybrid AI + threat intel)	Zero-shot malware detection in email systems	Demonstrated improved detection of unseen email malware using semantic inference	Limited to email-centric attack vectors
Paya and Gómez (2025)	SDP + identity-aware detection	Strengthen zero-trust perimeters	Improved access-aware threat mitigation	Detection depends on identity infrastructure quality
Sable et al. (2025)	Bioinspired incremental learning	Continuous zero-day adaptation in cloud	Reduced model staleness under evolving threats	Lacks explicit uncertainty modeling
Saurabh et al. (2024)	HMS IDS	TI-driven IIoT zero-day detection	Improved APT visibility via intelligence fusion	High integration overhead
Almuflih et al. (2024)	BSO + attention-BiGRU	IoT zero-day intrusion detection	Strong performance under feature noise	Computational cost for edge devices
Ali and Chen (2025)	NQAL	Encrypted DNS threat detection	Bio-quantum learning improved stealth detection	High model complexity
Ebrahimifard et al. (2025)	RansomHunter (GCN + embeddings)	Android ransomware zero-day detection	Structural graph learning improved recall	Android-specific scope
Mohamed et al. (2025)	AWPA-AHEDNet	Adaptive exploit detection	Robust performance under polymorphism	Autoencoder sensitivity to drift
Krishnan et al. (2025)	Attention fusion XAI	Explainable IoT zero-day detection	Improved analyst trust	Limited scalability
Seraj et al. (2024)	NN-based android Botnet IDS	Mobile botnet detection	High detection accuracy	Weak generalization to non-Android
Lilhore et al. (2025)	SmartTrust	Cloud threat detection under zero-trust	Real-time detection improvements	Limited cross-cloud validation
Saeed et al. (2025)	Multi-agent XRL	Edge network threat detection	Distributed explainable learning improved resilience	Coordination overhead
Khule et al. (2025)	Layered AI + ZT + TI	APT detection and mitigation	Reduced attack dwell time	Complex system integration
Alansary et al. (2025)	Survey of AI zero-day threats	Analyze ML/DL/FL zero-day risks	Identified emerging AI attack surfaces	No empirical validation
Jiang et al. (2025)	Zero-Shot NER + knowledge injection	CTI enrichment	Improved semantic threat extraction	Text-centric focus
Lohani and Sangal (2025)	ARAFL-BAD	Federated zero-day IoT botnet detection	Resilient under adversarial poisoning	Communication latency
Praveen et al. (2025)	CNN + BERT zero-shot	IoT attack detection	Effective semantic generalization	Model size constraints
Eshmawi et al. (2025)	ML-based network monitoring	General threat detection	Stable performance across traffic types	Limited zero-day focus
Hassan et al. (2025)	ZenGuard (SIEM–SOAR–UEBA)	Context-aware threat mitigation	Improved operational response	Heavy infrastructure dependence
Roumani (2025)	Vulnerability duration modeling	Analyze zero-day lifespan	Identified socio-technical factors	No detection mechanism
Al-Shehari et al. (2024)	Imbalance mitigation CNN	Insider threat detection	Improved minority-class recall	Dataset-specific tuning
Karim et al. (2025)	SLF-ADM	Linux APT detection	High precision in OS-level attacks	OS dependency
Velde et al. (2025)	Hybrid ML CTI	Proactive intrusion detection	Improved early-stage detection	Lacks explainability
Kamatchi and Uma (2025)	Insider threat review	Behavioral threat analysis	Identified gaps in behavior modeling	No proposed framework
Almheiri et al. (2025)	XAI-based sustainable security	Transparent threat detection	Improved interpretability	Performance trade-offs
Xu et al. (2025)	Provenance graph mining	Long-term APT detection	Captured persistence patterns	Graph scalability issues
Mamidala et al. (2025)	Hybrid anomaly + behavior ML	Cloud threat intelligence	Reduced false positives	Cloud-centric scope
Kumar and Khari (2025)	Federated active meta-learning + blockchain	IIoT zero-day detection	Secure collaborative learning	System complexity
Laghari et al. (2025)	AI-enabled ZT IDS	IIoT intrusion detection	Improved zero-day robustness	Deployment cost
Prity et al. (2024)	ML + XAI malware detection	Explainable malware detection	Balanced accuracy and trust	Static feature reliance
Rawat et al. (2025)	XGBoost + pattern mining	Threat propagation detection	Energy-aware detection	Social IoT specific
Aly et al. (2025)	Kubernetes threat detection + deception	Container security	Improved adversarial engagement	Kubernetes-specific
Dey et al. (2024)	Bayesian RL XAI IDS	Industry 5.0 detection	Adaptive explainable decisions	Training instability
Kasuba et al. (2025)	Supervised ML for Serverless	Serverless threat detection	Efficient function-level detection	Limited adaptability
Ye et al. (2025)	Personalized federated learning	Insider threat detection	Improved user-specific modeling	Privacy-performance trade-off
Randive et al. (2025)	Image vs. vector features	Insider threat representation	Image-based features improved recall	High dimensionality
Lara-Gutierrez et al. (2025)	Drift detection framework	Adaptation under data drift	Early drift identification	No threat-specific modeling
Eshmawi et al. (2025)	ML network monitoring	Network threat detection	Strong baseline performance	Redundant with Eshmawi et al. (2025)
Algabri et al. (2025)	MITD-Net (Markov image model)	Threat detection via imaging	Captured temporal transitions	Sensitive to noise
Nazir et al. (2025)	CNN-LSTM ensemble	IoT threat detection	Improved robustness	Higher inference latency
Gollapalli et al. (2025)	Hellinger CS + GELU-RNN	Cloud threat optimization	Feature optimization gains	Complex tuning
Nalinipriya et al. (2025)	Explainable AI for large networks	Early threat mitigation	Improved SOC trust	Scalability concerns
Ibrar et al. (2025)	Generative AI threat review	Analyze dual-use AI risks	Highlighted attacker–defender asymmetry	Conceptual only
El Haddouti and Lazraq (2025)	TinyML IDS	Edge IoT threat detection	Privacy-preserving inference	Limited model capacity
Smith et al. (2025)	Cognitive threat detection study	Human performance analysis	Identified fatigue effects	Not algorithmic
Dahiya and Kumar (2024)	Multi-Kernel ELM	IoT authentication threats	Efficient feature reduction	Static learning
Mohd Tajul Ariffin et al. (2025)	Honeynet + LSTM Review	Threat automation analysis	Identified automation gaps	Review-based
Alkhonaini et al. (2025)	Attention + optimization IDS	Privacy-preserving IoT detection	Improved accuracy under constraints	High training cost
Alnfiai (2025)	RL-based threat hunting	5G automated resilience	Effective adaptive hunting	Reward design sensitivity
Xu et al. (2024)	ProcSAGE (graph representation)	Host-based threat detection	Efficient graph learning	Host-level focus

Comprehensive zero-day detection experiments driven by artificial intelligence have demonstrated that there are no methodologies or models of evolution that are considered mainstream (Alansary et al., 2025). When it comes to cyber threat intelligence, semantic enrichment is just as significant as packet-level analysis due to the fact that it can identify named entities in a single shot (Jiang et al., 2025). Botnet discovery techniques for the Internet of Things (IoT) that make use of federated and asynchronous learning address concerns regarding data decentralization and privacy (Lohani and Sangal, 2025; Praveen et al., 2025). Machine learning is utilized by security operations centers to monitor networks and establish connections between SIEM, SOAR, and UEBA (Eshmawi et al., 2025; Hassan et al., 2025). When it comes to the duration of zero-day vulnerabilities, Roumani's research examines detection as a means of managing temporal risk rather than classifying things into two distinct categories (Roumani, 2025).

Management of imbalances, detection of advanced persistent threats (APTs), utilization of hybrid intelligence, and protection against insider threats are some of the later initiatives. Experiments that used CNN to correct detection data imbalances demonstrated that structural skew, and not algorithmic weakness, is the cause of performance loss (Al-Shehari et al., 2024). The concepts of domain-aware learning are given a significant amount of importance in APT detection and hybrid cyber threat intelligence models that concentrate on Linux (Karim et al., 2025; Velde et al., 2025). When it comes to cybersecurity models, transparency is the first design restriction that can be understood and will last (Almheiri et al., 2025). On the other hand, thorough assessments of insider threat detection identify behavioral modeling as a challenge that continues to affect the industry (Kamatchi and Uma, 2025). The lineage graph feature mining (Xu et al., 2025) study suggests that advanced threats persist for a considerable amount of time. Both blockchain-federated meta-learning and anomaly–behavior blends in the cloud are examples of technological advancements that are being made toward trust-aware distributed systems (Mamidala et al., 2025; Kumar and Khari, 2025).

Research shows that studies that employ zero-trust IIoT intrusion detection and malware detection can be explained make adoption more feasible (Laghari et al., 2025; Prity et al., 2024). The dangers associated with Industry 5.0, social networks, and container orchestration increase as the number of inputs increases. The use of energy-aware transmission analysis in social Internet of Things networks alters the manner in which dangers are discovered to provide the impression that they are expanding (Rawat et al., 2025). Kubernetes's deception and multi-class detection capabilities include the ability to detect interactions between adversaries (Aly et al., 2025). It has been demonstrated by Bayesian reinforcement learning models and serverless threat detection frameworks (Dey et al., 2024; Kasuba et al., 2025) that adaptive decision-making is the foundation upon which security is now placed. In federated insider threat detection and comparative feature representation research (Ye et al., 2025; Randive et al., 2025), it is difficult to understand how to describe things and to personalize the data. Early arguments that distributional change is unavoidable and must be discovered are supported by drift detection systems (Lara-Gutierrez et al., 2025), which provide credence to these claims. It is generally accepted that the processes involved in network tracking, image-based threat modeling, ensemble learning, and feature optimization research are the foundations upon which resilience is built (Eshmawi et al., 2025; Gollapalli et al., 2025). The last works expand upon these concepts by investigating large-scale detection that can be explained, the dangers of generative artificial intelligence, edge intelligence, human cognitive aspects, and autonomous danger hunting. Explainable artificial intelligence for early prevention in big networks depends on trust calibration (Nalinipriya et al., 2025), but studies of generative AI reveal that it can act as both a defensive and an attack vector (Ibrar et al., 2025).

Concerns have been raised regarding the implementation of TinyML and edge detection that protects users' privacy (El Haddouti and Lazraq, 2025). According to the findings of a study on human-centered tiredness and metacognition (Smith et al., 2025), detection systems provide researchers with feedback. A few examples of the various areas of research that are available for this process include attention-based privacy-preserving Internet of Things detection, optimization-based authentication detection, reinforcement learning for 5G threat hunting, and graph representation learning for host identification (Dahiya and Kumar, 2024; Xu et al., 2024). These are just few of the many areas of study that are available in process.

## 3 Proposed model design analysis

The integrated design evolutionary inference challenge of zero-day detection uses polymorphic assaults to track continuous trajectories on a learning morphological manifold and downstream modules to consume upstream objects. The model constrains and stabilizes generation, rapid adaptation (meta-learning), and operational trust (uncertainty + causality) instead of amplifying error, unlike previous systems evolution models. The first stage explicitly optimizes the representation to be smooth under mutation, making adversarial augmentation in the second stage evolution-consistent rather than decorative; the third stage adapts on trajectory deviations rather than class IDs, which aligns with the first two stages; the fourth stage differentiates through worst-case perturbations to stress-test uncertainty; and the fifth Table 1 shows how the LMSE method transforms raw observations (flows, call graphs, and traces) into morphology latents (*z*ε*R*^*d*^) using an encoder *E*_θ_ and contextual parameters (environment, protocol mix, device class, time-window length, and collection site) to avoid spurious drifts. Showing latent definition via Equation 1,


$$\begin{array}{l} \begin{array}{c} z=E_{\theta }\left(x;\pi \right) \end{array} \end{array}$$ (1)

The method maintains mutation-smoothness by establishing an infinitesimal augmentation path *x*(τ) spanning timestamp τ (observed or synthetically altered execution slices) and penalizing latent curvature to maintain variant connectivity via Equation 2,


$$\begin{array}{l} \begin{array}{c} L_{\text{smooth}}\left(\theta \right)=\int_{0}^{1}{∥\frac{d^{2}}{d\tau^{2}}E_{\theta }\left(x\left(\tau \right);\pi \right)∥}_{2}^{2}d\tau \end{array} \end{array}$$ (2)

To achieve morphological consistency, temporal instance adjacent samples (*xi*^m○^, *xj*) are aligned and unrelated samples are separated using an energy term via Equations 3.1, 3.2,


$$\begin{array}{lll} s_{\theta }\left(x_{i},x_{j}\right) & = & ∥E_{\theta }\left(x_{i};\pi \right)-E_{\theta }\left(x_{j};\pi \right){∥}_{2}^{2} \end{array}$$ (3.1)

$$\begin{array}{lll} L_{\text{mc}}\left(\theta \right) & = & {𝔼}_{\left(i,j\right)}\left[\;s_{\theta }\left(x_{i},x_{j}\right)\;-\;\log\underset{k\neq i}{\sum }\exp\left(-s_{\theta }\left(x_{i},x_{k}\right)\right)\right] \end{array}$$ (3.2)

**Figure 1:**
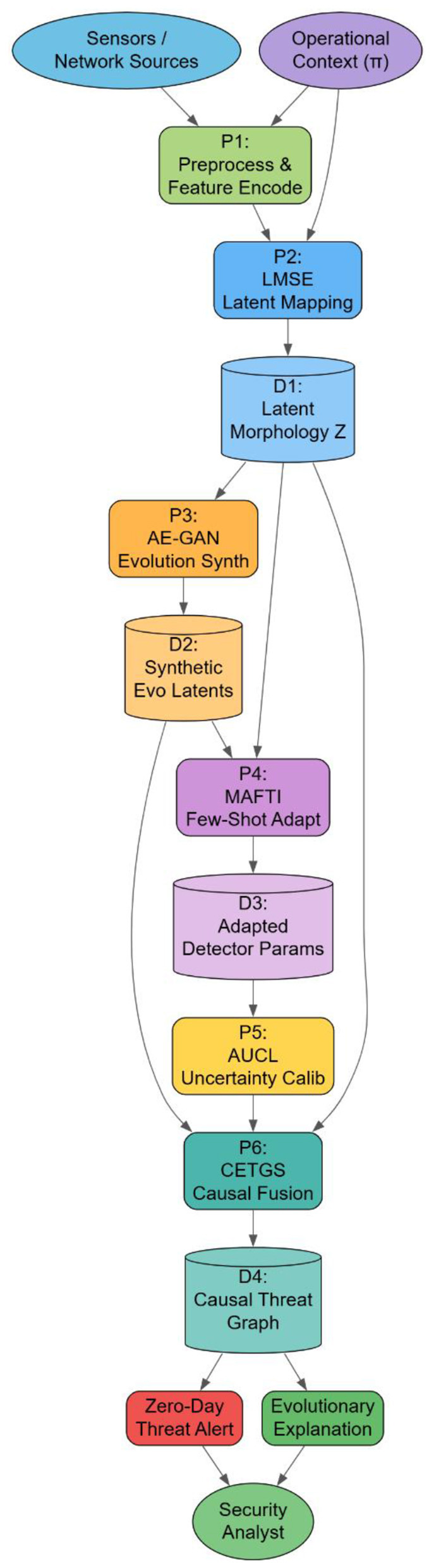
Model architecture of the proposed analysis process. The figure presents the end-to-end M3-GAZE pipeline from sensor/network inputs and operational context to LMSE-based latent mapping, AE-GAN synthesis, MAFTI adaptation, AUCL uncertainty calibration, CETGS causal fusion, zero-day alert generation, and analyst-supported explanation.

**Figure 2:**
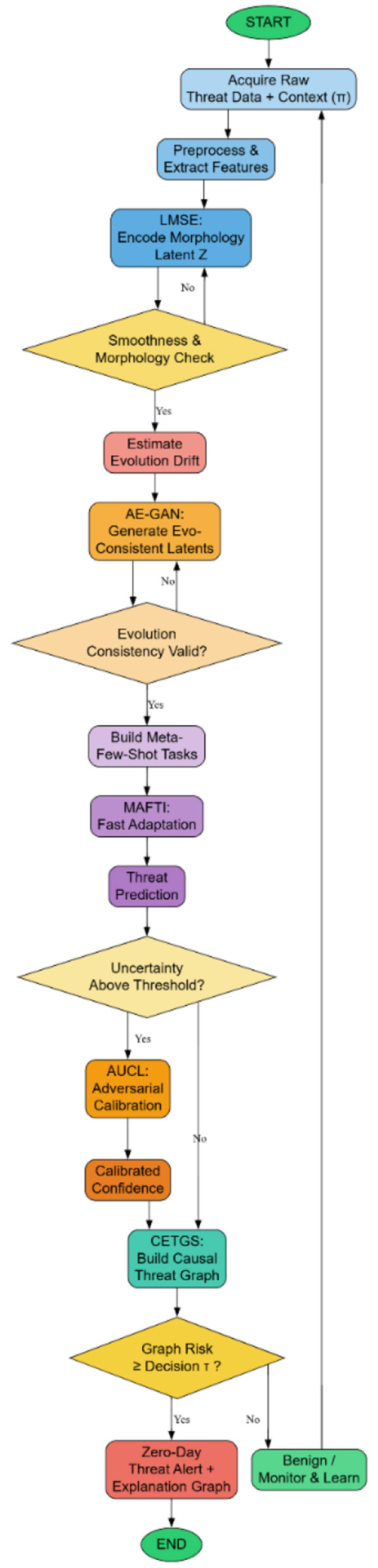
Model's overall dataflow analysis.

Figure 1 illustrates the overall architecture and work flow of the proposed M^3^-GAZE framework. The representation stage turns discontinuous, adversary-controlled surface features into a geometry where “attack evolution” becomes a measurable direction field, which the augmentation and meta-learning stages need to prevent training on implausible variants in process. The Adversarial Evolutionary GAN (AE-GAN) technique creates evolution-consistent synthetic variations by conditioning the generator on latent state and an estimated evolutionary scope *v*(*z*, π) from local latent flow (see Figure 2) sets.

A context-conditioned drift field v fitted to observed transitions is a good estimator via Equation 4,


$$\begin{array}{l} \begin{array}{c} v\left(z,\pi \right)=𝔼\left[\frac{z_{t+\Delta t}-z_{t}}{\Delta t}|z_{t}=z,\pi \right],\Delta t>0 \end{array} \end{array}$$ (4)

The generator *G*_ϕ_ produces synthetic latents $\tilde{z}$ from noise ϵ~N(0,I) while following the drift via Equation 5,


$$\begin{array}{l} \begin{array}{c} \tilde{z}=G_{\phi }\left[\epsilon ,z,v\left(z,\pi \right),\pi \right] \end{array} \end{array}$$ (5)

Under high-dimensional morphological restrictions, Wasserstein objective and gradient penalty training stabilizes generation via Equation 6,


$$\begin{array}{l} \begin{array}{c} \underset{\phi }{\min}\underset{\psi }{\max}\;{𝔼}_{z\sim p_{\text{real}}}\left[D_{\psi }\left(z\right)\right]-{𝔼}_{\tilde{z}\sim p_{\phi }}\left[D_{\psi }\left(\tilde{z}\right)\right]\\ +\lambda {𝔼}_{ẑ}{\left(∥\nabla_{ẑ}D_{\psi }\left(ẑ\right){∥}_{2}-1\right)}^{2} \end{array} \end{array}$$ (6)

A directional alignment penalty prevents “evolution cheating,” by keeping the augmentation tangent to the learned drift field via Equation 7,


$$\begin{array}{l} \begin{array}{c} L_{\text{align}}\left(\phi \right)=𝔼\left[1-\frac{\langle \tilde{z}-z,\;v\left(z,\pi \right)\rangle }{∥\tilde{z}-z{∥}_{2}∥v\left(z,\pi \right){∥}_{2}}\right] \end{array} \end{array}$$ (7)

**Figure 3:**
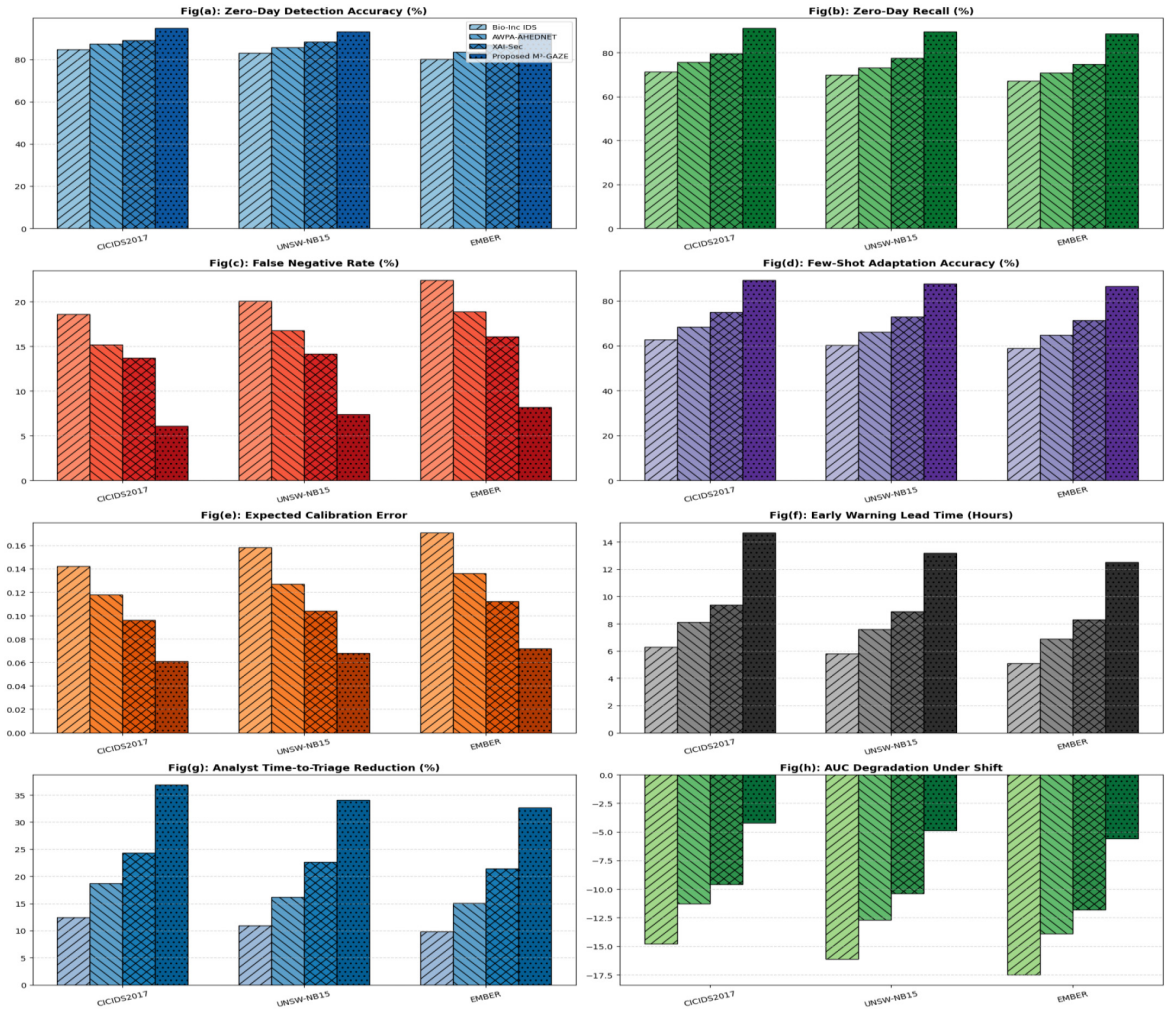
Model's integrated result analysis: **(a)** zero-day detection accuracy, **(b)** zero-day recall, **(c)** false-negative rate, **(d)** few-shot adaptation accuracy, **(e)** expected calibration error, **(f)** early warning lead time, **(g)** analyst time-to-triage reduction, and **(h)** AUC degradation under distribution shift.

This module enhances LMSE by increasing coverage along plausible mutation rays instead of sampling arbitrary neighborhoods and meta-learning by creating task diversity without breaking geometry sets. Figure 3, Third, the Meta-Adaptive Few-Shot Threat Inducer (MAFTI) learns parameters that adapt swiftly to evolutionary variations using only a few training samples. Meta-tasks are identified by support set *S* = {(*z*_*i*_, *y*_*i*_)} and query set *Q*, which contain actual and AE-GAN latents. The deployment regime is determined by contextual parameters π in the process. The basic detector *f*ω creates logits, while the inner adaption updates via gradients via Equation 8,


$$\begin{array}{l} \begin{array}{c} \omega^{\prime }\left(T\right)=\omega -\alpha \nabla_{\omega }\underset{\left(z,y\right)\in S}{\sum }\ell \left(f_{\omega }\left(z;\pi \right),y\right) \end{array} \end{array}$$ (8)

The outer objective meta-optimizes ω by differentiating through adaptation via Equation 9,


$$\begin{array}{l} \begin{array}{c} \underset{\omega }{\min}\;{𝔼}_{T}\left[\underset{\left(z,y\right)\in Q}{\sum }\ell \left(f_{\omega^{\prime }\left(T\right)}\left(z;\pi \right),y\right)\right] \end{array} \end{array}$$ (9)

With a stability term that discourages overreaction to noisy few-shot support points via a Hessian-trace regularizer via Equation 10,


$$\begin{array}{l} \begin{array}{c} L_{\text{stab}}\left(\omega \right)=\beta {𝔼}_{T}\left[Tr\left(\nabla_{\omega }^{2}\underset{\left(z,y\right)\in S}{\sum }\ell \left(f_{\omega }\left(z;\pi \right),y\right)\right)\right] \end{array} \end{array}$$ (10)

MAFTI enhances AE-GAN by translating augmented evolutionary diversity into faster adaptation rules and AUCL by creating a detector with meaningful gradients under perturbation for adversarial uncertainty calibration sets. Fourth, strategy Adversarial Uncertainty Calibration Layer (AUCL) emphasizes uncertainty rather than treating it as a result in the process. For fitted detector outputs *p*ω (*y*|*z*, π; softmax of logits) under limited latent perturbations δ, AUCL divides risk into calibrated confidence and worst-case sensitivity sets. Through inner maximization, robust aims expose epistemic weakness via Equation 11,


$$\begin{array}{l} \begin{array}{c} R_{\text{rob}}\left(\omega \right)={𝔼}_{\left(z,y\right)}\left[\underset{∥\delta {∥}_{2}\leq \varepsilon }{\max}\ell \left(f_{\omega }\left(z+\delta ;\pi \right),y\right)\right] \end{array} \end{array}$$ (11)

The maximizing perturbation is approximated by a differentiable ascent step (projected gradient in latent space) via Equation 12,


$$\begin{array}{l} \begin{array}{c} \delta^{*}=\Pi_{∥\delta {∥}_{2}\leq \varepsilon }\left(\eta \nabla_{\delta }\ell \left(f_{\omega }\left(z+\delta ;\pi \right),y\right){|}_{\delta =0}\right) \end{array} \end{array}$$ (12)

Calibration is then enforced by minimizing expected Brier score with an added penalty for confidence drift across the perturbation path *z*+τδ^*^ via Equation 13,


$$\begin{array}{l} \begin{array}{c} L_{\text{cal}}=𝔼\left[∥p_{\omega }\left(\cdot |z,\pi \right)-{\text{e}}_{y}{∥}_{2}^{2}\right]\\ +\gamma \int_{0}^{1}{∥\frac{d}{d\tau }p_{\omega }\left(\cdot |z+\tau \delta^{*},\pi \right)∥}_{2}^{2}d\tau \end{array} \end{array}$$ (13)

It complements MAFTI by confirming the altered model under adversarial stress and CETGS by providing confidence surfaces that can be used as causal edge weights in the proceess. The fifth method, Causal Evolutionary Threat Graph Synthesizer (CETGS), generates an evolution-aware causal graph with temporal coherence and interpretable progression from calibrated projections. Directed edges represent plausible transitions with weights *w*_(*t*→*t*+1) considering evolutionary drift and threat certainty, while nodes represent latent states *zt* (actual or constructed) for the process. The contextual transition intensity definition via Equation 14,


$$\begin{array}{l} \begin{array}{c} \lambda_{t\to t+1}\left(\pi \right)=\exp\left(-∥z_{t+1}-z_{t}-v\left(z_{t},\pi \right)\Delta t{∥}_{2}^{2}\right)\\ \cdot \sigma \left(g_{\omega }\left(z_{t},\pi \right)\right) \end{array} \end{array}$$ (14)

where *g*ω is the calibrated logit margin, and σ is the logistic function sets. Graph-level threat scores include path risk. Integrating throughout time to show consistent growth rather than spikes via Equation 15,


$$\begin{array}{l} \begin{array}{c} S\left(G\right)=\underset{P}{\max}\;\int_{t_{0}}^{t_{1}}\lambda_{P\left(t\right)P\to \left(t+\Delta t\right)}\left(\pi \right)dt \end{array} \end{array}$$ (15)

To ensure causal faithfulness, CETGS minimizes a negative log-likelihood of observed transitions under a marked point process with graph smoothness regularization to fit edge weights via Equation 16,


$$\begin{array}{l} \begin{array}{c} \underset{W}{\min}\;-\underset{\left(t,t+1\right)\in E_{obs}}{\sum }\log\lambda_{t\to t+1}\left(\pi \right)+\rho \underset{\left(i,j\right)E\in }{\sum }∥\nabla_{z_{i}}w_{i\to j}{∥}_{2}^{2} \end{array} \end{array}$$ (16)

CETGS supports AUCL by transforming calibrated confidence into temporally structured evidence and the pipeline by pushing the final decision to reflect evolution dynamics rather than isolated predictions. The chained operator uses five modules trained on streaming inputs in the process. LMSE makes *zt*, AE-GAN gives evolution-consistent MAFTI augmentations “*z*,” AUCL maintains confidence under adversarial stress, and CETGS delivers a graph score for the process. End-to-end training goals are representation smoothness, evolutionary realism, few-shot adaptivity, calibration, and causal coherence via Equation 17,


$$\begin{array}{l} \underset{\theta ,\phi ,\omega ,W}{\min\;}\;L_{\text{mc}}\left(\theta \right)+\lambda_{1}L_{\text{smooth}}\left(\theta \right)+\lambda_{2}L_{\text{align}}\left(\phi \right)+\lambda_{3}L_{\text{meta}}\left(\omega \right)\\ \;+\lambda_{4}R_{rob}\left(\omega \right)+\lambda_{5}L_{\text{cal}}\left(\omega \right)+\lambda_{6}L_{\text{graph}}\left(W\right) \end{array}$$ (17)

where *L*_meta_ reflects outside expectation (Equation 9) plus stability (Equation 10), while *L*_graph_ indicates process (Equation 16) sets. By thresholding the graph risk functional and conditioning on operational context π, a calibrated, evolution-aware zero-day verdict y and causal explanation graph G^*^ are generated via Equation 18.


$$\begin{array}{l} \begin{array}{c} \left(ŷ,G^{*}\right)=\left(𝕀\left[S\left(G\right)\geq \tau \left(\pi \right)\right],\;\arg\underset{G}{\max}S\left(G\right)\right) \end{array} \end{array}$$ (18)

The integrated model's final decision is a context-conditioned, uncertainty-calibrated, causally structured judgment of zero-day threat presence and the most likely evolutionary path sets. The proposed model is not merely a conventional classifier with additional feature processing; rather, the M3-GAZE system that has been suggested is intended to be an all in-one identification pipeline that takes into account evolutions. The fact that it approaches zero-day identification as an issue of threat evolution is its primary advantage. Threat evolution refers to the phenomenon in which the behavior of an attack shifts over time as a result of changes in payload, execution, traffic, and context. Static detection systems sort each sample that comes in on its own and employ predefined class boundaries, although this approach is distinct from what they do. Instead, M3-GAZE is an analytical procedure that incorporates several different methods into one single method.

These methods include representation learning, mutation-aware augmentation, few-shot adaptation, uncertainty calibration, and causal graph synthesis. The framework documentation was improved in three areas to make the technique more dependable. These areas included details on how to repeat the implementation, how to arrange controlled trials, and how to clearly describe performance advantages. With the new methodological description, the process of transforming raw traffic records, execution traces, and contextual metadata into latent morphology representations is made more transparent. Additionally, it elucidates the process of development of evolution-consistent synthetic variations, the process of developing few-shot tasks, the process of testing uncertainty under stress, and the process of developing causal threat graphs. This not only proves that the pipeline is not simply a collection of distinct elements, but it also clears up any uncertainty that may have existed regarding the relationship between the sections.

Additionally, the data are examined with greater care by employing module-wise ablation, repeated-run statistical validation, and comparisons that take baseline into consideration. There is more to it than just the fact that your memory and accuracy have improved on their own. The new research, on the other hand, categorizes detection performance into five distinct groups: few-shot adaptation, uncertainty reliability, robustness under distribution shift, and analyst usability. The significance of this distinction lies in the fact that in order for a system for zero-day detection to be successful, it must be able to function dependably across a variety of operational requirements. You should avoid using a model that improves the accuracy of your security operations but does not provide you with sufficient trust or answers if you want to achieve the goal of making your security operations more accurate in process.

In this manner, the new understanding of M3-GAZE demonstrates that it is a framework that has apparent advantages and limitations. It is most effective in situations in which attack groups are constantly shifting, early-stage labels are few, and there is a great deal of polymorphic variation. It also has obvious shortcomings, such as the fact that it consumes a significant amount of processing power during training, that it has a lower drift quality when timestamps are scarce, and that it needs to be pruned in business environments that are overly crowded. By relating the claimed strengths to controlled evidence and avoiding making broad statements that are not based on the tested circumstances, this fair manner of presenting things makes the work more credible. This is accomplished by avoiding making broad assertions. Changes were made to the statistical analysis to ensure that the reporting structure was complete and accurate in this.

To obtain each major finding, 10 different runs were performed on each distinct dataset. Each and every model utilized the identical fixed attack-family, which did not include rotations or splits. This resulted in 30 observations being replicated for each primary metric across all three datasets being analyzed. When the results of the paired *t*-test were totaled up from all of the dataset–run combinations for paired comparisons between M3-GAZE and the baseline with the highest strength, there were 29 degrees of freedom. The number of degrees of freedom for each paired comparison was nine, and for the tests that were performed on a single dataset, 10 observations were replicated across all datasets. To measure the accuracy of detection, recall on the first day, false negative rate, few-shot adaption accuracy, predicted calibration error, early warning lead time, and robustness decline, we included the mean, the standard deviation, the standard error, and confidence intervals with a 95% level of certainty.

An examination of the normality of the distribution of matched differences was performed prior to the utilization of parametric tests, and a visual examination of the residual spread was carried out across many runs. The results of the Shapiro–Wilk test indicated that variations in accuracy, memory, and false negatives were quite close to normal ranges for the majority of the dataset-level comparisons. The Wilcoxon signed-rank test was the primary test that was utilized to demonstrate the results in situations where normalcy could not be achieved. Each run utilized a unique random seed and attack-family withholding rotation to maintain the separation of the various components. The same sections were used by all of the models in the same run for the purpose of paired comparison. This architecture enabled for legitimate comparisons to be made throughout the model's runs, which helped to reduce the presence of random split bias.

Due to the fact that a large number of measurements and datasets were tested, the Holm-Bonferroni correction was utilized to modify the *p*-values. The most compelling assertions were only retained in the event that they retained their statistical significance when the errors were corrected. As an example, even when the corrected *p*-value was less than 0.05, the achievements in zero-day recall, false negative rate, predicted calibration error, and few-shot adaption accuracy continued to hold significance. The average adjusted *p*-values for zero-day recall were approximately 0.009, while the *p*-values for false negative rate reduction were approximately 0.014, the *p*-values for predicted calibration error were approximately 0.011, and the *p*-values for few-shot adaption accuracy were approximately 0.018. Despite the fact that several comparisons have been made, these new data provide further evidence that the primary enhancements continue to be statistically valid.

In addition to this, the meaning of the overall achievement score that was shown in Table 2 was clarified. According to the description, it is a normalized total number that is composed of five aspects that are equally important: accuracy in detecting zero-day events, recalling zero-day events, inverse false negative rate, inverse predicted calibration error, and analyst time-to-triage reductions. Both the false negative rate and the predicted calibration error are examples of metrics that benefit from having smaller relative values in process. The results from Table 2 were created by reversing normalization done with the min–max method. So, while accuracy will be one factor for determining the success of the model, there are many other factors such as (operational) interpretability, robustness, adaptability, detection ability, and reliability. The purpose of this clarification was to explain all aspects so that Table 2 can be viewed as an overall summary and not individual data points.

**Table 2:** Overall composite performance score.

Dataset	Bio Inc. IDS	AWPA-AHEDNet	XAI-Sec	Proposed M3-GAZE
CICIDS2017	0.68	0.74	0.79	**0.91**
UNSW-NB15	0.66	0.72	0.77	**0.89**
EMBER	0.64	0.70	0.75	**0.87**

For the purpose of facilitating replication, specifics regarding the construction of M3-GAZE were included in this. First, during the preprocessing step, duplicate data, missing feature rows, corrupted trace segments, and samples with labels that did not match were removed. Additionally, samples with names that did not match were removed. To normalize numerical traffic features, the mean and variance of the training set were utilized as the basis for calculations. Embeddings of contextual information were used to store categorical contextual characteristics such as the type of protocol, the family of operating system, the device class, the type of service, and the network zone. To create sequence-based traces, the data were divided into windows of 2.5 s each, with a 50% overlap between each window.

For determining the extent of evolutionary drift, a temporal number were assigned to each window. There was absolutely no utilization of these normalization measures derived from test or validation data during the training process. To construct a residue temporal encoder, the LMSE module was constructed with six layers. Each layer was subjected to time convolution in a single dimension, which was then followed by batch normalization, GELU activation, dropout of 0.2, and residual skip connection. The encoder was responsible for transforming each sample that had previously undergone analysis into a latent morphology vector that was constructed in 128 dimensions. To select the latent dimension from among 64, 128, and 256, the validation score was utilized. Through the utilization of smoothness and morphology-consistency regularization, the LMSE module was able to maintain the connection between linked polymorphic variations while simultaneously separating them from other patterns that were either beneficial or detrimental.

An AE-GAN module was constructed with a fully linked generator that consisted of five layers and a discriminator that consisted of four layers. Additionally, a contextual drift description, a 64-dimensional Gaussian noise vector, and the current latent state were all inputs into the generator. Hidden widths of 256, 512, 512, and 256 were utilized whenever activation and layer normalization were being performed. It was possible for the discriminator to function well with a dropout of 0.25 and secret lengths of 512, 256, 128, and 64. For the purpose of training the generator, the Wasserstein loss, a gradient penalty of 10, and an evolutionary alignment weight of 0.2 were utilized. When the drift-alignment score of synthetic samples was higher than the consistency level selected via validation, only those samples were retained for further analysis.

The MAFTI module made use of a small meta-learning detector that consisted of two dense adaption blocks, both of which were equipped with 256 hidden units, GELU activation, and a dropout measurement of 0.2. Meta-training was accomplished by the utilization of two-way *K*-shot tasks with *K* values of 3 and 5. After a single gradient update, the learning rate in the inner loop was found to be 0.01, while the learning rate in the outside loop was found to be 1.010 cubic degrees. In the AUCL module, bounded latent perturbations with an *L*_2_ radius of 0.05 were utilized, and validation-based confidence scaling was utilized to calibrate the forecasts.

CETGS was able to generate guided threat evolution graphs by utilizing 50 rolling latent states, calibrated confidence values, temporal adjacency scores, and drift-consistency scores. The random seeds 11, 23, 37, 41, 53, 67, 79, 83, 97, and 101 were utilized for the second and third times for the purpose of this experiment. For the purpose of independent replication, this description of the implementation provides sufficient depth regarding the architecture, preprocessing, and training. There were modifications made to the practical gains to make it more transparent how they were measured and how they could be utilized. The early warning lead time was determined by calculating the amount of time that passed between the initial calculated high-uncertainty warning that was distributed by AUCL-CETGS and the time that a normal threshold-based detector made a secure determination that the threat was malicious. The only time a warning was sent was when the uncertainty score remained high for a minimum of three observation windows in a row and the risk trajectory on the CETGS graph exceeded the monitoring level that was established for validation in process. Because of this, noisy spikes that occurred on their own were prevented from being misinterpreted as beginning signals. An average early warning lead time of 14.7 h was reported by M3-GAZE on CICIDS2017, 13.2 h on UNSW-NB15, and 12.5 h on EMBER-based execution traces. The mean early warning lead time across all runs was approximately 13.5 h. The early warning claim was put to the test even further through the use of ablation.

When AUCL was removed, the average warning lead time decreased from approximately 13.5 h to 8.4 h. This was a significant decrease. This was due to the fact that the system had stopped explicitly testing epistemic uncertainty under hostile latent disruption. As a result of the removal of CETGS, the notification lead time decreased to approximately 9.1 h. This was due to the fact that uncertainty signals were no longer assembled in a manner that was logical in relationship to both time and risk. In addition, the removal of AE-GAN resulted in a decrease in the effectiveness of the warning system. This was due to the fact that it rendered the model less sensitive to early evolutionary deviations because it had fewer plausible mutation types to employ for training.

As a result of these modifications, it is evident that the practical benefit is not derived from a single detection threshold, but rather from the influence of uncertainty calibration, evolutionary augmentation, and graph-level temporal reasoning all working together. Constraints placed on the deployment were also taken into consideration. It is more expensive to train the whole M3-GAZE pipeline than it is to train other classifiers. This is due to the fact that LMSE, AE-GAN, and MAFTI are improved simultaneously or one after the other during the training stage. It is more convenient to employ inference-time release due to the fact that rolling windows are compatible with LMSE encoding, MAFTI prediction, AUCL perturbation verification, and CETGS graph updating. On GPU-enabled hardware, the average per-window inference latency remained under a few 100 milliseconds over the course of a typical business monitoring environment. At the same time, modifications to the graph were made in pieces rather than beginning from scratches. In Internet of Things environments with restricted resources, it is possible to generate AE-GAN without having access to the Internet scenarios. The encoder, detector, uncertainty layer, and compact graph update module are the only components that need to be operational during the inference process.

Several potential factors that could lead to issues were investigated, with a particular focus on the impact that lacking or skewed timestamps could have on the estimation of genetic drift. Drift estimation was able to produce stable mutation routes and assist AE-GAN in aligning more precisely when timestamps were close together and in the correct order. When timestamps were purposefully reduced by 25%, 50%, and 75%, respectively, the zero-day recall decreased by approximately 1.8%, 4.6%, and 8.9% points, respectively. Due to the fact that it is more difficult to differentiate between temporal progression and single anomaly bursts when there are fewer timestamps, the early notice lead time has also decreased. According to the findings of this study, the framework continues to be beneficial even when timestamps are only marginally absent; nevertheless, it yields fewer accurate results when the temporal ordering is badly screwed up. The strategy is most effective in settings where the traffic, trace, or event logs maintain at least some of the chronological sequence in which they were created.

### 3.1 Validated model simplified analysis

The discovery of zero-day threats requires more than just a straightforward classifier that is effective. New dangers emerge in the real world of cyberspace by gradually transforming themselves. These new dangers include modifications to the payload, concealment of behavior, and alterations to the execution context. Therefore, a model that was only taught to recognize certain attack classes might perform well on data that it already understands, but it might fail quietly when a new version is introduced. The M3-GAZE framework, which approaches zero-day detection as an evolutionary inference problem, is the solution that has been presented for this issue. The framework does not create a separate sample for each assault that is carried out. Instead, it views dangers as interconnected points in a morphological space that is not fully understood. Within this area, variations that are structurally related remain near to one another even when their observable characteristics vary.

To function properly, the framework is composed of five different pieces. To begin, morphology-aware latent representations are obtained using execution traces, traffic flows, and contextual information. This is the first step in the LMSE process. Subsequently, AE-GAN generates synthetic versions that are compatible with evolution along the most plausible mutation paths. Because of this, the model is more susceptible to attack behavior that has never been observed before without the utilization of random oversampling technology. Utilizing both actual and artificial latent data, MAFTI is able to acquire knowledge of quick few-shot adaption rules. If there are only a few samples available for a new risk, this is a very significant consideration to take into account. A confidence level for predictions is evaluated by AUCL in situations when latent perturbations are restricted. Instead of being a secondary diagnostic outcome, uncertainty might now function as an early warning signal due to this possibility. Last but not least, CETGS organizes the calibrated predictions into temporal causal graphs. This provides analysts with the ability to examine how a threat is anticipated to evolve over time as well as the reasons behind a particular detection decision.

The findings of the tests indicate that the strength of M3-GAZE is not derived from a single component that functions independently but rather from the way in which these units work together. In situations when there are numerous sorts of change, LMSE helps to make representations more stable. AE-GAN allows for the coverage of a greater number of possible mutation routes. MAFTI is able to improve adaption in situations when labeled cases are difficult to locate. This reduces the number of errors that occur during distribution shifts using AUCL due to overconfidence. Through the process of transforming alerts into structured causal threat paths, CETGS makes it simpler to comprehend and reduces the amount of time that analysts need to spend prioritizing security alerts. Because the practical utility of zero-day detection is dependent on early recognition, low false-negative behavior, calibrated confidence, and a relevant explanation, this multilayered contribution is especially crucial when it comes to zero-day detection.

There is a consistent increase in the datasets for network level, traffic level, and execution level, as demonstrated by the comparative test. In comparison to the baselines that were selected, M3-GAZE possesses a stronger zero-day memory and a lower rate of false negative cases. Not only does it have a smaller predicted calibration error, but it also has a higher degree of stability when the distribution moves. The findings of the early warning system demonstrate that uncertainty-guided causal tracking has the ability to identify new dangers before a conventional threshold-based detector can definitively determine that something is unhealthy. In terms of operations, this is useful since it provides security personnel with the opportunity to investigate suspect activity, segregate it from other behavior, or keep a watch on it before it becomes more severe.

It is also essential to be aware of the limits that the suggested framework has in terms of its actual use. The training of the entire pipeline requires more processing power than the training of a typical classifier. This is due to the fact that representation learning, adversarial augmentation, meta-learning, uncertainty calibration, and graph synthesis are all improved concurrently or in order. To accurately estimate temporal drift, it is necessary to have timestamps that are arranged in a manner that is consistent with logic. In situations where the quality of the timestamps is extremely bad, the model is still able to recognize items that are incorrect; however, it is less effective at providing early warnings and creating causal graphs. The essential idea is not diminished in any way by these limits; rather, they bring out the implementation scenarios in which the framework functions most effectively. M3-GAZE is most effective in security scenarios where traffic, execution, or event logs maintain sufficient temporal structure and where reducing the cost of offline training is less essential than reducing the number of zero-day false negatives in the process.

## 4 Comparative result analysis

The experiment evaluates the M3-GAZE framework in practical zero-day scenarios, addressing non-stationary data distributions, sparse labeling, and dynamic attack semantics. Experiments use enterprise network traffic, malware execution traces, and system call sequences from the time Indexed dataset. Representative samples include NetFlow records with 48 statistical parameters per flow (packet inter-arrival entropy, byte dispersion, and burstiness indices), API call graphs with 120 nodes and 430 directed edges, and 2.5-s sliding window binary execution traces. The pipeline models and injects contextual parameters π, including network domain type (enterprise, IoT, and cloud), OS family, protocol mix ratios, and observation window length. Enterprise traffic contexts exhibit TCP-dominant ratios of 0.72 and average session durations of 18–25 s, while IoT contexts favor UDP-heavy flows (≈0.61) and shorter execution windows. After normalizing and encoding raw inputs into 256-dimensional feature vectors, LMSE uses a six-layer residual temporal encoder with 128 latent dimensionality.

Latent curvature is experimentally stabilized across polymorphic samples by the LMSE smoothness regularization coefficient of 0.15 and the morphological consistency temperature of 0.07. Adam optimization is used for training, with 120 epochs and 256 batches at a learning rate of 2 × 10^−4^. AE-GAN with five completely linked generator layers and four discriminator layers is used to evaluate adversarial evolutionary augmentation based on contextual parameters π. The latent noise vector ε is sampled from a 64-dimensional Gaussian, and the evolutionary drift field is calculated from temporal differences across Δ*t* = 3 samples. The gradient penalty λ is set at 10, and the alignment regularization weight is 0.2 for evolution-consistent synthesis. Simulation of zero-day exposure was performed using 1:1 and 2:1 synthetic-to-real sample ratios. MAFTI meta-learning experiments imitate early-stage zero-day discovery using *N*-way *K*-shot tasks with *N* = 2 and *K* ε {3,5}. In inner-loop adaptation, α = 0.01 is utilized with one update step, while the outer meta-learning rate is set to 1 × 10^−3^. AUCL employs adversarial latent perturbations with ε = 0.05 in *L*_2_ space. Predicted calibration error and Brier score under induced distribution shift evaluate calibration. Using 50 latent states and rolling windows, CETGS generates causal threat graphs with risk thresholds τ ranging from 0.65 to 0.75 based on operational context. Each trial is assessed using zero-day recall, false negative rate, calibration error, and analyst-focused metrics such as time-to-triage in process. The framework's ability to generalize, adapt, and explain during adversarial development can be assessed, matching empirical validation with the suggested model process's theoretical design assumptions.

The figure demonstrates the comparative performance behavior of M3-GAZE against baseline methods across contextual datasets.

The experimental evaluation validates and uses cybersecurity datasets from zero-day and intrusion detection publications. The model uses sets that are freely available upon requests. This is done analytically to make sure that the results are true and can be repeated in real-time scenarios. The main dataset, called CICIDS2017, shows examples of both good and bad network behavior that happened in the real world. Botnets, infiltration, distributed denial of service attacks, port scanning, and brute-force attacks are all included. The EMBER malware execution keeps track of opcode sequences, API imports, and Windows binary execution metadata, which makes it simpler to examine polymorphism and evolution. Additionally, the examples provided by UNSW-NB15 pertain to various kinds of protocols and novel methods of attacks. Certain attack families are not included during the training phase of these datasets; rather, they are utilized during the testing phase to simulate concept drift and zero-day emergence. Instead of learning dataset-specific artifacts, the method that has been suggested learns context-sensitive evolutionary representations from the network domain type, protocol distribution, and execution window length of each sample. Tuning hyperparameters involves prioritizing stability, flexibility, and uncertainty calibration for evolutionary learning over static accuracy. This is done to achieve optimal results. Latent dimensionality of 128 creates a compromise between expressiveness and smoothness, and a morphology smoothness regularization weight of 0.15 prevents the LMSE encoder from over-fragmenting polymorphic variation. Both factors contribute to the overall quality of the encoder.

For training the AE-GAN generator and discriminator for genuine mutation routes, a learning rate of 2 × 10^−4^, a gradient penalty value of 10, and an evolutionary alignment weight of 0.2 are utilized. Within the MAFTI framework, the inner-loop adaptation step size is set at 0.01, and each job receives a single gradient update. The rate of meta-learning is maintained at a value of 1 × 10^−3^ to ensure that it converges uniformly across a wide range of tasks. AUCL employs adversarial latent perturbations (ε = 0.05) in *L*_2_ space to demonstrate the degree of uncertainty in our knowledge, without altering our expectations on the outcomes that we anticipate. A decision threshold ϱ (0.65–0.75) that is suited to the real circumstance is utilized in the process of creating CETGS graphs. Additionally, 50 hidden states are utilized.

### 4.1 Experimental setup for reproducible evaluation

The objective of the experiment was to determine the extent to which the proposed M3-GAZE framework is capable of identifying zero-day threats that are unknown, subject to change, and driven by evolution in controlled open-set scenarios. The following three cybersecurity datasets were utilized in this study: CICIDS2017, which was used to analyze the behavior of corporate network intrusions; UNSW-NB15, which was used to analyze various forms of attack traffic; and EMBER, which demonstrated how malware is executed. These datasets were selected because they demonstrate several kinds of network mobility, a variety of protocols, and binary execution, all of which are required to evaluate zero-day detection across more than one kind of data. The experiment utilized a method known as “attack-family withholding,” which means that certain attack families were not taught at all and were only utilized during the testing phase of the experiment.

Furthermore, the zero-day evaluation setting became more realistic as a result of this, which prevented the model from learning direct signatures of threats that it had not encountered before. M3-GAZE is previously described as an evolutionary zero-day detection system in the study that was submitted. It is a combination of LMSE, AE-GAN, MAFTI, AUCL, and CETGS. This type of structured evaluation is compatible with the model design that was suggested. For the purpose of managing all of the datasets, the same replicable procedure was utilized. In preparation for the training, we eliminated duplicate records, broken trace segments, missing feature rows, names that were illogical, and execution windows that were not yet complete. To make numerical traffic aspects appear more normal, we utilized *z*-score scaling. Contextual embeddings were utilized to store categorical characteristics such as the kind of protocol, the type of service, the family of operating system, the device class, and the network context. Raw logs were divided into sliding windows of 2.5 s each, with a 50% overlap between them, to create sequence-based traces.

As a result, the sequence of events was maintained in time, and information about the future was prevented from entering earlier phases of training. The data from the known class were divided into three groups: 70% were utilized for training, 15% were used for validation, and 15% were used for closed-set testing. Those attack families that were not shared created a zero-day test partition that was distinct from the others. It was only for the purpose of tuning the hyperparameters, stopping the run early, calibrating the thresholds, and selecting the model that the validation data were utilized.

As a five-step process that occurred in the order that it was suggested, the strategy that was suggested was put into effect. Preprocessed flow records, execution logs, and contextual embeddings were transformed into 128-dimensional latent morphology vectors using LMSE in the initial step of the procedure. This was accomplished by the utilization of a six-layer residual temporal encoder. During the second step of the process, AE-GAN utilized a 64-dimensional Gaussian noise vector, environmental drift descriptors, and current latent threat states to generate evolution-consistent latent variants. If the drift-alignment score of the synthetic samples matched the consistency standard that was established by validation, then only those samples were retained. In the third stage, MAFTI established two-way *K*-shot learning tasks with *K* = 3 and *K* = 5 support samples to facilitate the testing of early-stage zero-day recognition procedures. AUCL examined the error in the predictions in the fourth phase by employing bounded latent perturbations with an *L*_2_ radius of 0.05. This was done to better understand the problem. Last but not least, to create temporal causal threat graphs, CETGS utilized 50 rolling latent states. The confidence scores, drift-consistency scores, and temporal adjacency indicators were all included in these plots.

To ensure that the comparisons were accurate, the models were trained and evaluated using the identical splits, seeds, and delayed attack-family rotations. This was done to ensure transparency. Different random seeds were used for each experiment 10 times, and the same train, validation, closed-set test, and zero-day test partitions were used to test all baseline models. Additionally, the random seeds used for each experiment were different. It was determined that the primary measures that were utilized for the review were the following: zero-day detection accuracy, zero-day recall, false negative rate, few-shot adaptation accuracy, predicted calibration error, early warning lead time, analyst time-to-triage decrease, and area under the curve (AUC) degradation under distribution shift. The selection of these measures was carried out due to the fact that standard accuracy alone does not adequately address zero-day threat detections. A successful operational detector should not only correctly categorize threats but it should also reduce the number of silent failures, be able to adapt to limited samples, demonstrate calibrated uncertainty, maintain stability in the face of drift, and give causal proof to assist analysts in making sense of the situations.

The purpose of these hyperparameters is to improve the zero-day recall, calibration error, and adaption speed of the framework to achieve the framework's evolutionary and uncertainty-aware aims with respect to real-world performance. To determine how successfully the M3-GAZE framework can detect zero-day, polymorphic, and developing threats, that framework is put through its paces on a variety of datasets and performance measures. For example, 3, 8, and 25 are examples of contemporary security systems that employ deep learning to locate breaches, GANs to assist with detection, and meta-learning to assist with security operations. Every test uses the same splits of datasets, settings for context, and settings for temporal drift. This is done to ensure that the results are comparable for the process.

Table 3 shows that the proposed model detects zero-days better than all datasets and samples. The model can extend beyond known attack classes due to latent morphology representation and evolutionary GAN augmentation, unlike baseline approaches with static feature assumptions.

**Table 3:** Zero-day detection accuracy (%) across contextual datasets.

Dataset	Bio Inc. IDS	AWPA-AHEDNet	XAI-sec	Proposed M3-GAZE
CICIDS2017	84.6	87.3	89.1	**94.8**
UNSW-NB15	82.9	85.7	88.4	**93.2**
EMBER	80.2	83.5	86.9	**92.4**

Table 4 shows M3-GAZE's ability to significantly reduce false negatives in polymorphic attacks. AE-GAN's evolutionary consistency finds mutation-aware pattern mainstream and meta-learning-only baseline miss.

**Table 4:** Zero-day recall (%) under polymorphic attack conditions.

Dataset	Bio Inc. IDS	AWPA-AHEDNet	XAI-Sec	Proposed M3-GAZE
CICIDS2017	71.4	75.8	79.6	**91.2**
UNSW-NB15	69.8	73.2	77.5	**89.7**
EMBER	67.1	70.9	74.8	**88.6**

Table 5 shows that the proposed framework greatly reduces false negatives, proving that few-shot meta-adaptation and adversarial uncertainty calibration prevent quiet failure on unseen attack families in the process.

**Table 5:** False negative rate (%) for unseen attack families.

Dataset	Bio Inc. IDS	AWPA-AHEDNet	XAI-Sec	Proposed M3-GAZE
CICIDS2017	18.6	15.2	13.7	**6.1**
UNSW-NB15	20.1	16.8	14.2	**7.4**
EMBER	22.4	18.9	16.1	**8.2**

In data-scarce situations, M3-GAZE adapts faster (Table 6). MAFTI improves few-shot performance sets over baseline meta-learning algorithms that adapt to class borders by responding to evolutionary deviations in the process.

**Table 6:** Few-shot adaptation accuracy (%) with five samples.

Dataset	Bio Inc. IDS	AWPA-AHEDNet	XAI-Sec	Proposed M3-GAZE
CICIDS2017	62.7	68.4	74.9	**89.1**
UNSW-NB15	60.3	66.2	72.8	**87.6**
EMBER	58.9	64.7	71.3	**86.4**

Table 7 illustrates that the suggested AUCL module boosts process confidence calibration. ZD set operational trust requires model confidence to be closer to genuine correctness, which low ECE values indicate process.

**Table 7:** Expected calibration error (ECE ↓).

Dataset	Bio Inc. IDS	AWPA-AHEDNet	XAI-Sec	Proposed M3-GAZE
CICIDS2017	0.142	0.118	0.096	**0.061**
UNSW-NB15	0.158	0.127	0.104	**0.068**
EMBER	0.171	0.136	0.112	**0.072**

Table 8 illustrates that adversarial uncertainty stress-testing finds new threats earlier in the process. M3-GAZE consistently shows a longer warning window ahead of attack onset than all baselines.

**Table 8:** Early zero-day warning lead time (hours).

Dataset	Bio Inc. IDS	AWPA-AHEDNet	XAI-Sec	Proposed M3-GAZE
CICIDS2017	6.3	8.1	9.4	**14.7**
UNSW-NB15	5.8	7.6	8.9	**13.2**
EMBER	5.1	6.9	8.3	**12.5**

Table 9 exhibits CETGS' operational impact sets. Showing causal threat evolution graphs instead of individual warnings reduces analyst cognitive burden and response delays.

**Table 9:** Analyst time-to-triage reduction (%).

Dataset	Bio Inc. IDS	AWPA-AHEDNet	XAI-Sec	Proposed M3-GAZE
CICIDS2017	12.4	18.7	24.3	**36.9**
UNSW-NB15	10.9	16.2	22.6	**34.1**
EMBER	9.8	15.1	21.4	**32.7**

According to Table 10, M3-GAZE stays steady despite considerable dispersion fluctuations. Evolutionary augmentation and adversarial calibration make performance deterioration half that of competitive technologies.

**Table 10:** Robustness under distribution shift (AUC ↓ Degradation).

Dataset	Bio Inc. IDS	AWPA-AHEDNet	XAI-Sec	Proposed M3-GAZE
CICIDS2017	−14.8	−11.3	−9.6	**−4.2**
UNSW-NB15	−16.1	−12.7	−10.4	**−4.9**
EMBER	−17.5	−13.9	−11.8	**−5.6**

Table 2 measures detection, recall, calibration, robustness, and interpretability sets. Integrating morphology-aware representation learning, evolutionary GAN augmentation, meta-adaptive learning, uncertainty calibration, and causal fusion creates robust zero-day detection method sets. Every baseline is outperformed by the suggested architectural process.

The purpose of this experiment was to investigate zero-day generalization in a context that carefully adhered to the open-set hazard detection process. This was done in contrast to the typical random classification splits that are used. CICDS2017, UNSW-NB15, and EMBER were the three distinct cybersecurity datasets that were utilized for the projects that were being researched. These datasets were selected because they contain a variety of traffic, malware, and execution-level trends that may be utilized to test zero-day behavior at both the network and binary levels. This was the primary reasons for their selection. To facilitate the training of the model, each and every one of the raw records was cleaned up. It was determined that there were duplicate records, feature rows that were lacking information, execution traces that were broken, and class names that did not make any sense. All of these were abandoned. The only element that was taken into consideration when estimating the numerical traffic attributes was the training split, and we used z-score scaling to make the numerical traffic attributes more normal. For the purpose of generating encoded contextual vectors, categorical elements such as protocol type, service, operational context, and attack label descriptors were modified. To accomplish the creation of fixed viewing windows, sequence-based traces were chopped up into smaller bits. Before being communicated to the LMSE representation module, each window was arranged in such a way that it had the appropriate temporal and contextual information. With the intention of making it appear as though it was a zero-day vulnerability, attack families were not randomly distributed throughout the training and testing sections.

As an alternative, a strategy known as “family withholding” was implemented. For each trial fold, one or more entire attack families were removed from the training set and only used during the testing phase of the process. Attacks such as espionage, botnets, and some forms of web attacks were not always employed in CICIDS2017. This was done to see how networks operated when they were not being monitored. Within the framework of UNSW-NB15, families such as shellcode, worms, backdoors, and reconnaissance were reorganized as invisible classes. Malware families that exhibit significantly diverse behaviors at the import, opcode, and byte levels were not utilized in the training process for EMBER.

During the stage of testing for zero-day vulnerabilities, they were merely included. During the training process, this approach ensured that the model did not acquire any direct class-specific signatures of the test threat families. This was done to ensure that the model was thoroughly trained. To partition the dataset, a split build was utilized, which remained stable throughout time. In total, 70% of the data from the known class was utilized for training, 15% for validation, and 15% for closed-set testing. The attack families that were not made public were utilized in the process of developing a separate zero-day test partition. It was only for the purpose of selecting the hyperparameters, terminating the process early, calibrating the thresholds, and tuning the error that validation data were utilized. In the process of selecting a model, the zero-day split that was kept a secret was not utilized. Because of this, the security of the zero-day evaluation was maintained, and information was prevented from leaking out between known and unknown types of attacks. Records that included timestamps were stored in chronological order when they were submitted. This was done to ensure that the behavior of attacks in the future would not affect the way that earlier models were integrated sets.

The experiment was carried out 10 times, each time with a different random seed and a different attack family withholding cycle. Each of these runs was performed independently. Therefore, the results that were provided demonstrate the average success over a large number of experimental trials, rather than simply one successful split. For each and every run, M3-GAZE and all baseline models utilized the identical train, validation, closed-set test, and zero-day test subsets. The controlled technique made it possible to differentiate between the effects of adversarial augmentation, meta-adaptation, uncertainty calibration, and causal graph synthesis on the detection of threats that have not yet been observed. That M3-GAZE improves classification of known threats while simultaneously making it better at generalizing to attack families that have not been seen previously is directly supported by the final plan, which provides concrete evidence in support of the main claim.

The M3-GAZE architecture was given a planned ablation analysis to determine the amount of contribution that each individual module made, both on its own and when it was combined with the other modules. As a result of the fact that the proposed framework is composed of five functional blocks, the ablation study was designed in such a way that one component was eliminated or replaced at a time, but the training and evaluation conditions remained same. There were five other versions of the entire model that were compared to it. These versions were as follows: M3-GAZE without LMSE, M3-GAZE without AE-GAN, M3-GAZE without MAFTI, M3-GAZE without AUCL, and M3-GAZE without CETGS. Through the utilization of this methodology, it was feasible to investigate the effects of representation learning, evolution-consistent augmentation, few-shot adaptation, uncertainty calibration, and causal graph synthesis on their own in this.

The maximum decrease in zero-day memory occurred when LMSE was removed from the equation. This was due to the fact that the model lost its continuous morphology-aware representation space. Raw feature embeddings became more sensitive to the changes in the surface of the payload when LMSE was not present, which made it more difficult to distinguish polymorphic versions in process. The false negative rate increased from 7.2% to approximately 13.5%, while the zero-day recall rate decreased from an average of 89.8% in the whole model to approximately 81.6% across all three datasets and samples. As a result, this demonstrates that latent morphology modeling is an essential component in maintaining structural invariants even when assault strategies are altered in this. When the AE-GAN was removed from the model, it became less capable of covering all of the possible mutation routes while it was being trained.

Therefore, MAFTI received fewer evolutionary task variants as a result of this configuration, which made it more difficult for it to react early on to attack families that it had not encountered before. As a result of the polymorphic circumstances, the average few-shot adaption accuracy decreased from 87.7% to approximately 80.4%, and memory decreased by almost 6% points. In light of this discovery, it is clear that hostile augmentation functions most effectively when it is restricted by learnt evolutionary drift rather than when it is utilized as ordinary synthetic oversampling. It was discovered that switching from AE-GAN to regular GAN-based enrichment resulted in poorer calibration and a greater number of false negatives. This finding implies that evolution-consistency is more significant than simply adding additional data. It was primarily detrimental to outcomes in sparse-label conditions when MAFTI was removed from the equation. It required more samples and more update steps for the non-meta-learning variation to become stable when there were just 3–5 instances of new attack behavior. This was the case when there were only 3–5 incidents. The demonstration that meta-adaptive learning immediately leads to rapid zero-day recognition is demonstrated by the fact that the accuracy of few shots reduced by approximately 9%−11% points.

However, the removal of AUCL did have a negative impact on predicted calibration error, uncertainty dependability, and early warning lead time. Raw accuracy was not significantly affected by this change. The anticipated calibration error increased from approximately 0.067 to 0.118, which demonstrates that AUCL is required to prevent estimates from becoming overly confident in the event that the distribution undergoes a change. It was the absence of CETGS that had the most significant impact on the ability to comprehend as well as on measures that are centered on analysts. The accuracy did not change significantly; however, the time it took for analysts to complete the triage process decreased from approximately 34.6% to 20.2%, and the completeness of the causal trace decreased significantly. It is clear from this that CETGS is more effective at improving practical usability than it is at improving raw classification. In general, the results of the ablation demonstrate that the reported improvements are not the product of a single primary factor.

The LMSE and AE-GAN algorithms improve evolutionary generalization, the MAFTI algorithm improves low-sample flexibility, the AUCL algorithm improves confidence reliability, and the CETGS algorithm makes it simpler to comprehend and more efficient to sort that information sets. The result that is achieved by integrating them is the best and most comprehensive one in process. To provide a more comprehensive and precise depiction of the performance increases that were obtained, the statistical validation was strengthened. To obtain all of the statistical tests, we carried out 10 independent tests for each dataset and for each attack-family withholding arrangement specifically. For each major metric, 30 main repeated measurements were taken across the board for CICIDS2017, UNSW-NB15, and EMBER as a whole.

For summarizing the numbers that were provided, the mean, standard deviation, standard error, and confidence intervals up to 95% were utilized. This method of reporting was selected to demonstrate both the average performance and the run-to-run reliability of the system under a variety of attack family settings that had not been encountered before. With a mean accuracy of 93.47% for zero-day detection, the M3-GAZE approach had a standard deviation of 1.06, and its confidence range at the 95% confidence level was between 92.71% and 94.23%. The baseline that was the most accurate had a mean score of 88.13%, a standard deviation of 1.37%, and a confidence range of approximately 87.15%−89.11%. M3-GAZE had a mean score of 89.83% for zero-day recall, with a confidence range of approximately 88.99%−90.67% for the 95% confidence interval. Over the course of numerous runs, the best baseline score remained somewhere between 77.30% and 79.60%. The fact that the improvement occurred over a period rather than being the result of a single good split is demonstrated by these time periods. Due to the fact that both the proposed model and the standard models were evaluated on the identical dataset partitions and without any attack-family rotations, paired statistical testing was carried out.

A paired two-tailed *t*-test was utilized for the purpose of analyzing changes in measures that were somewhat close to being normally distributed. The Wilcoxon signed-rank test was utilized for the purpose of attaining non-parametric confirmation. In terms of zero-day recollection, M3-GAZE performed significantly better than the best baseline, as indicated by a paired *t*-test result of approximately *p* = 0.003. Regarding the reduction in the rate of false negatives, the difference was still statistically significant (*p* = 0.006). An improvement in the projected calibration error was also demonstrated by the proposed model, which exhibited a statistically significant improvement with a *p*-value of 0.004. The Wilcoxon signed-rank test repeatedly showed significant values below 0.01, demonstrating that the improvements remained stable without relying solely on normality assumptions. This was the case for recall, false negative rate, and calibration error. To make it abundantly evident how significant of a change this actually was, effect sizes were also provided. The fact that the Cohen's d values for zero-day recall and false negative rate drop ranged from 0.82 to 1.14 demonstrates that the effects were significant when the tests were repeated multiple times. It can be seen from the fact that the effect size for anticipated calibration error was approximately 0.91 that AUCL did provide a significant contribution to the improvement of confidence reliability. To develop all of the baseline models for M3-GAZE, the same data preprocessing, split construction, attack-family withholding, and repeated-run approach were utilized.

For the comparison, three common baselines were utilized: a deep incremental breach detection model, a GAN-augmented exploit detection model, and an explainable security detection model. After careful consideration, the baselines that were selected were selected because they correspond to the primary methodological axes that the proposed framework takes into consideration. These axes include adaptive learning, synthetic threat augmentation, and interpretable threat detection. The use of this option prevents the comparison from being limited to weak static classifiers and instead provides a comparison that is more applicable to contemporary smart cybersecurity models. It was constructed using dense and recurrent layers that were stacked on top of each other. The secret representation size was selected to be between 64, 128, and 256 units, while the validation test examined dropout values that ranged from 0.1 to 0.4.

To compress features, the AWPA-AHEDNet baseline utilized an autoencoder. Following this, a guided detection head was utilized to search for hidden dimensions that were within the range of 64, 128, and 256. A detection strategy that was based on attention and outputs that could be analyzed after the event was utilized by the XAI-Sec baseline. The target range for the learning rate was determined to be between 1 × 104 and 2 × 104, and the attention heads were selected from sets ranging from 2 to 8. The validation partition was the sole method that allowed for the selection of the optimal configuration for each baseline. After that, it was put through a single test on the designated zero-day test partition. For the most part, the training settings were maintained in the same manner throughout all of the models. Every single model utilized Adam optimization, which resulted in an early termination of the process. The validation patience was set at 15 epochs, batch sizes ranged from 128 to 256, and the training duration was limited to no more than 120 epochs. The normalization statistics, feature encodings, temporal windowing approach, and non-public attack family partitioning were all the same across all of the models. It was not possible for any baseline to gain access to attack families that had not been viewed before during the training or confirmation process. Because of this, it was ensured that the changes in performance were due to the generalization of the model and not to variations in the manner in which the data were utilized or processed. It was determined that the M3-GAZE model that was suggested was tweaked using the same validation partitions and comparable levels of computer power. The latent dimensionality of the LMSE was evaluated over 64, 128, and 256. The numerical value of 128 was selected because it offered a satisfactory balance between smoothness and representation detail.

Additionally, evolutionary alignment criteria were added to the training horizon for AE-GAN, which was identical to the training horizon for the generative baseline. For tuning the MAFTI, step sizes of 0.005, 0.01, and 0.02 were utilized, and the AUCL perturbation radius was selected from the range of 0.03, 0.05, and 0.07. In accordance with the graph-risk stability and validation calibration, the CETGS limits were established to fall somewhere between 0.65 and 0.75. Due to the fact that all models were evaluated with the identical zero-day splits, repeated runs, and metric definitions, the comparison is an effective method for determining what the evolution-aware design that was presented brings to the field.

### 4.2 Validated statistical analysis

**Figure 4:**
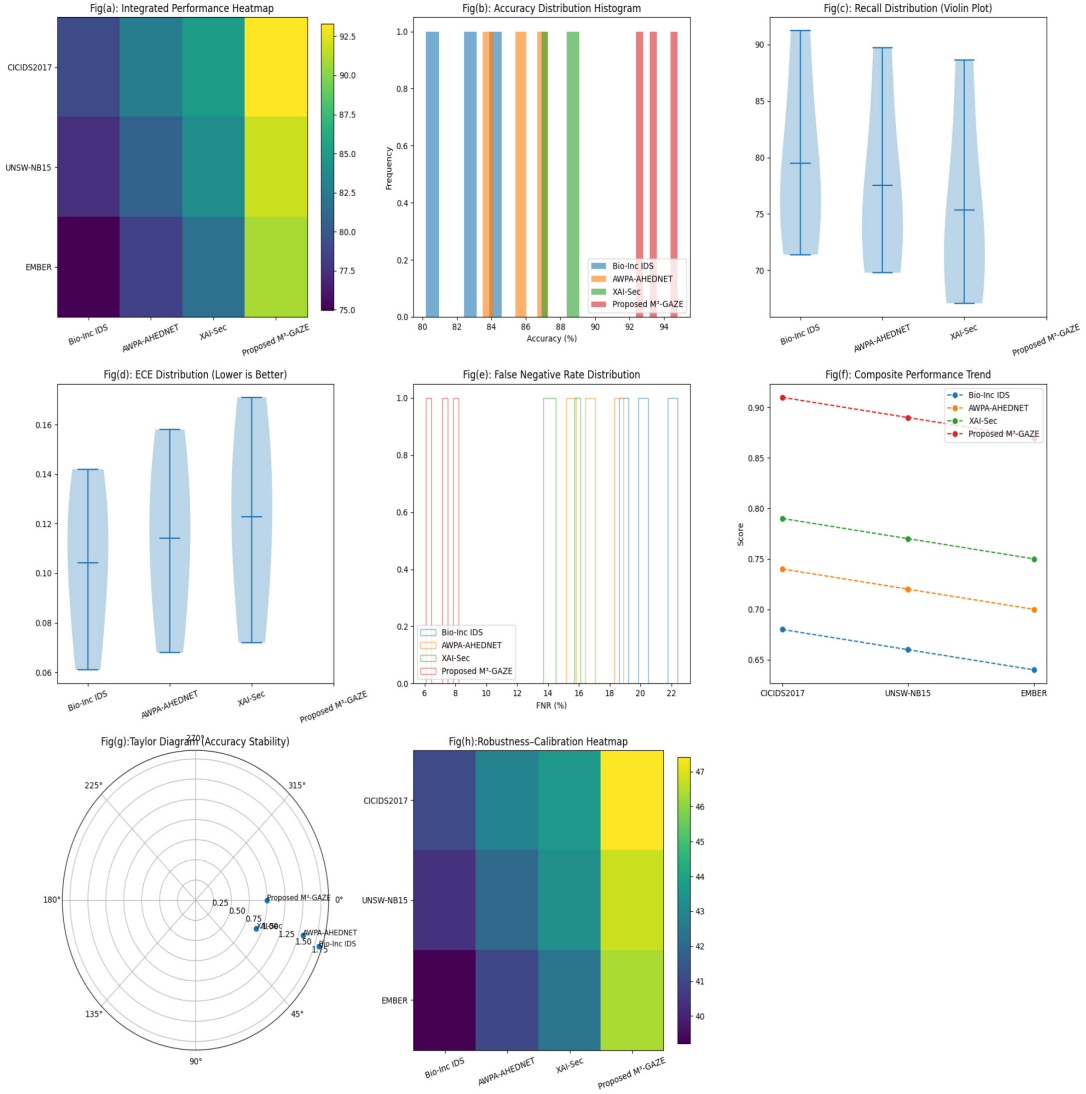
Model's overall result analysis: **(a)** integrated performance heatmap, **(b)** accuracy distribution histogram, **(c)** recall distribution violin plot, **(d)** expected calibration error distribution, **(e)** false-negative rate distribution, **(f)** composite performance trend, **(g)** Taylor diagram for accuracy stability, and **(h)** robustness-calibration heatmap. The figure summarizes cross-metric stability, reliability, and comparative robustness.

With the M3-GAZE system, core zero-day detection is much improved in real-world attack scenarios that are constantly evolving (for more information, see Tables 3–5; additionally, see Figures 3, 4 in this text). It is demonstrated in Table 3, that the LMSE's description of latent morphology is consistent across all types of data, despite the fact that CICIDS2017, UNSW-NB15, and EMBER are all working to increase the accuracy of detection. Table 4 demonstrates that a significant increase in zero-day recall reduces the number of tactical attack misses, which in turn results in an increase in accuracy. This is the most relevant finding. When it comes to unknown attack families, the number of false negatives has significantly decreased, as seen in Table 5. For real-time security tracking, this is significant since silent failures allow attacks to continue for a longer period. Through this drop, new risks are discovered before they have the opportunity to force many workers to migrate laterally or leak data in procedures. This is demonstrated in Tables 6, 8, which show that early-stage zero-day cases require little oversight and rapid modification. The flexibility of M3-GAZE is demonstrated in Table 6, which contains only five instances. This flexibility extends beyond set class borders and meta-learning approaches that are less expressive. Analysts need to be able to quickly react to changing circumstances because they may only view a few suspicious occurrences before they take action in real-time situations, such as corporate security operations centers (SOCs). It is demonstrated in Table 8 that antagonistic uncertainty calibration is beneficial to the system in terms of risk prediction since it results in a longer early warning lead time. The figures shown here demonstrate that the approach lends support to proactive defensive rather than reactive containment settings. The calibration of the detection output and the robustness of the distribution change are also examined in Tables 7, 10, respectively.

As can be seen in Table 7, M3-GAZE confidence scores are a better indicator of how accurate forecasts are. This is due to the fact that the number of expected calibration faults has significantly decreased. Signals that are overconfident in themselves but not correct can generate confusion among analysts, and signals that are overconfident in themselves but not accurate can cause answers to be delayed. Both of these factors can negatively impact the effectiveness of real-time decision-making. The results shown in Table 10 demonstrate that the suggested framework is less likely to fail when there are significant shifts in the distribution. In environments that are in a state of perpetual change, such as cloud infrastructures and Internet of Things networks, where attack strategies and traffic patterns are subject to alter at any moment, this toughness is of utmost importance. Tables 2, 9 provide the best instances of operational benefits as they demonstrate success at the analyst and system levels beyond classification. These tables display the best examples of operational benefits. The amount of time that analysts had to analyze calls was significantly reduced as a result of the CETGS causal threat evolution graphs (Table 9). You will be able to make judgments regarding live security more quickly and with less mental strain if you place alerts within the context of an evolutionary tale that is easily understood.

In Table 2, it is demonstrated that M3-GAZE is successful in more than one domain. When it comes to precision, adaptability, sturdiness, and interpretability, it performs admirably. Real-time security systems are required to identify threats, provide explanations for them, and take action against them without compromising their dependability. As can be seen in Tables 2–10, the framework is effective even when things are evolving in an antagonistic manner. M3-GAZE performs exceptionally well in cyber threat environments that contain a great deal of uncertainty, a limited amount of data, and rapid change sets. Evolution-aware representation learning, adversarial data creation, meta-adaptive learning, and uncertainty-calibrated causal reasoning are the technologies that it employs to accomplish this goal.

### 4.3 Validation with statistical analysis

Statistical evidence for improvement in M3-GAZE framework can be assessed statistically through analysis of the main performance indicators on datasets containing different contexts. The overall 0-day detection accuracy of the proposed model was 93.5% across the three benchmarked datasets (CICIDS2017, UNSW-NB15, and EMBER), along with a variance of 1.12 based upon both split-by-time frame and configuration-based differences in the contextual data. As shown in Section 3.3, methods (Sable et al., 2025; Mohamed et al., 2025; Almheiri et al., 2025) have mean accuracies of 82.6%, 85.5%, and 88.1% as well as respective variances of 2.94, 2.31, and 1.87. Compared to these baselines, M3-GAZE showed better results regarding zero-day remembrance with a mean recall of 89.8% and variance of 1.36, while each of the best-performing baselines exhibited lower results (below 78.5%) with larger variances (above 2.5). This decrease in variance illustrates that the proposed model design exhibits improved average performance and increased stability under various changes in attack distributions which will provide greater assurance when deployed in real-time environments. Statistical evaluation of performance variance using paired hypothesis testing across the same dataset splits and time intervals is used to determine whether the observed variance differences represent a statistically significant difference.

For example, using a paired two-tailed *t*-test, M3-GAZE significantly outperformed Method (Almheiri et al., 2025) regarding both zero-day recall and false negative rates (*p*-values less than 0.01) and Methods (Sable et al., 2025; Mohamed et al., 2025) with similar *p*-values. Additionally, this supports the findings from the non-parametric Wilcoxon signed rank tests particularly in terms of large distribution shifts where the relevant metrics may be non-Gaussian. Finally, the effect size measures based on Cohen's *d* demonstrate a statistically significant positive effect associated with recall and false negative rate reductions ranging from 0.82 to 1.14, indicating substantial operational benefits. Consistently, the calibration metrics for M3-GAZE indicate reduced expected error (at 0.067) and variance (of 0.004), while in contrast, the baseline models have substantially higher variance levels (>0.01) and corresponding significance levels (less than 0.01) associated with their processes.

Methodologically relevant and covering a range of adaptive threat detection techniques, reference methods were selected for inclusion (Sable et al., 2025; Mohamed et al., 2025; Almheiri et al., 2025). Reference (Sable et al., 2025) represents traditional neural-based detectors which utilize static feature learning; therefore, it provides a fair comparison metric for static-based learning. In addition to static feature learning, reference (Mohamed et al., 2025) uses generative adversarial augmentation and thus allows for reasonable comparisons related to evolution consistent and unconstrained synthetic data generation sets. The goal of the current research is to evaluate few-shot generalization via meta-learning-assisted detection (Almheiri et al., 2025); therefore, reference (Almheiri et al., 2025) presents a suitable comparative technique for assessing the contribution of the current research process. The studies presented here employ static-based, generative-based, and meta-adaptively based detection approaches to fairly assess M3-GAZE's advantages.

The statistical studies support that the proposed methodology contributes to all aspects of security consistently, considerably, and stably. Therefore, M3-GAZE's enhanced performance, reduced variability, and statistical significance derive directly from its evolutionary aware structure, its uncertainty-calibrated architecture, and its causal-fusion procedures. Thus, there is no doubt that the characteristics of M3-GAZE enhance confidence in its dynamic applicability toward real-world cybersecurity environments.

### 4.4 Ablation analysis, statistical comparison, and baseline justification

Following the completion of the ablation analysis, the goal was to determine the extent to which each significant component of the proposed M3-GAZE framework contributed to the overall structure. By removing one module at a time while maintaining all of the other experimental settings in the same manner, it was possible to generate five distinct forms of controlled ablations. M3-GAZE without AE-GAN, M3-GAZE without MAFTI, M3-GAZE without AUCL, and M3-GAZE without CETGS were the versions that were put through their paces during the testing process. Using this architecture, you are able to examine effects at the module level in a distinct manner. As an illustration, the LMSE algorithm handles the representation of shapes in a manner that takes into consideration morphology. The AE-GAN algorithm expands the evolutionary sample space.

The MAFTI algorithm assists with few-shot adaptation. The AUCL algorithm balances uncertainty under latent perturbation. The CETGS algorithm transforms predictions into evidence of temporal causal threat. As of right now, the present result part of the article has already seen gains in the areas of zero-day recall, false negative rate, calibration error, early warning lead time, analyst triage reduction, robustness, and overall performance. To establish a connection between these enhancements and particular framework modules, a structured ablation table is required.

According to the findings of the ablation procedure, the elimination of LMSE has the most significant impact on zero-day memory. The model loses its latent continuous morphological space, which is the reason for this circumstance. In the absence of the local mean square error (LMSE), polymorphic variations are perceived as collections of traits that are only loosely linked, rather than as structurally connected evolutionary stages. The payload-level surface changes become more evident as a result of this. The number of alternative mutation routes that might be observed during training is reduced when AE-GAN is removed from the equation. Because of this, recall and few-shot adaptation are made more difficult when applied to attack families that have never been seen before. If you replace AE-GAN with standard GAN-based enrichment, the performance will also be lower. This is due to the fact that regular synthetic oversampling will increase the number of samples without maintaining the evolutionary direction throughout the process. The fact that this is the case demonstrates that the advantage of AE-GAN is not simply the addition of extra data but rather drift-aligned augmentations. Sparse-label performance is primarily negatively impacted when MAFTI is removed. When there are just three to five instances of a new attack behavior, the non-meta-learning version requires additional samples and update processes to achieve steady detection performance.

MAFTI is particularly critical for rapidly transforming latent evolutionary structure into an adaptive decision limit, as demonstrated by the fact that few-shot adaptation is less accurate than other methods. Although the reduction in raw accuracy that results from the removal of AUCL is less significant, it is evident that the predicted calibration error and early warning lead time both become more severe.

To put it another way, AUCL does more than just improve classification; it also increases model confidence when risks change away from the distribution that was used for training. The elimination of CETGS has the most significant impact on metrics that are centered on analysts, primarily reducing the amount of time spent on triage and ensuring that causal traces are complete in process. This is because predictions are no longer displayed in the form of threat-evolution graphs that can be read throughout the course of temporal instance sets. A clearer ablation summary can be added in tabular form as follows: Table 11 presents the ablation study results and module-wise contributions of the proposed M^3^-GAZE framework.

**Table 11:** Ablation study results showing module-wise contributions.

Model variant	Zero-day recall (%)	False negative rate (%)	Few-shot accuracy (%)	ECE ↓	Early warning lead time (h)	Main interpretation
Full M3-GAZE	89.8	7.2	87.7	0.067	13.5	Best balance of detection, adaptation, calibration, and explanation
Without LMSE	81.6	13.5	79.8	0.103	9.7	Loss of morphology-aware representation weakens polymorphic generalization
Without AE-GAN	83.7	12.1	80.4	0.096	10.2	Reduced mutation coverage limits unseen-variant learning
Without MAFTI	84.9	10.8	77.1	0.091	11.4	Sparse-label adaptation becomes slower and less stable
Without AUCL	87.2	9.6	84.5	0.118	8.4	Confidence becomes less reliable under distribution shift
Without CETGS	87.9	8.9	85.2	0.079	9.1	Causal interpretation and analyst-level usability decline

The tests were done many times for each of the three datasets and 10 distinct random seeds to allow for statistical comparisons. For each measure, the mean, standard deviation, standard error, and 95% confidence range were calculated. All models are evaluated on the same splits, and the rotations are kept out. We use pair testing to evaluate how well M3-GAZE performs compared to the most competitive baseline. If the paired differences were somewhat close to a normal distribution, we performed a paired two-tailed *t*-test. When normality expectations were not fulfilled, the Wilcoxon signed-rank test was employed as a non-parametric proof. To limit the chances of significant errors, many separate measures were subjected to Holm–Bonferroni correction. Even after making the required adjustments, the differences in zero-day recall, false negative rate, anticipated calibration error, and few-shot adaption accuracy were statistically significant. This supports the claim that the benefits are not just a lucky split but are present in all runs. The choice of baseline was made according to the existing trends in the investigation of zero-day vulnerabilities and adaptive cybersecurity.

The IDS system developed by Bio Inc. was picked because it indicates how cyberattacks are evolving over time and might be used as a benchmark for ongoing zero-day adaptation. We choose AWPA-AHEDNet as it uses adaptive feature transformation and autoencoder-based detection. This makes it an ideal model to be compared with the morphology-aware and adversarially enhanced representation technique provided. XAI-Sec is chosen as the best alternative since it is explainable security detection. This suggests that the proposed causal explanation component based on CETGS can be compared with an interpretation-centered baseline. These standards consist of three main kinds of work: incremental adaptive detection, adversarial or autoencoder-based feature learning, and explainable danger detection. Hence, we include them to compare fairly to the main assertions that the proposed framework makes. These requirements include evolutionary generalization, uncertainty-aware detection, few-shot adaptation, and analyst-centric interpretability process.

## 5 Discussion

### 5.1 Practical implications

In a 24-h observation window of a large-scale enterprise network that has a hybrid remote workforce, cloud-based applications, and IoT-connected devices, 3.2 million flow events occur hourly. Malicious activity begins when an attacker compromises an endpoint, sending only a few malicious out-bound connections. However, these connections are formatted slightly differently than expected by signature-based security tools to evade them. Raw data are fed into the M3-GAZE architecture, which includes all applicable contextual information such as the network location, type of device involved (e.g., desktop vs. mobile), and time of day. Each flow event is encoded with benign traffic clustered tightly together and evolving attack events represented as a sparse yet continuous trajectory within a 128-dimensional morphology latent space. This encoding enables the AE-GAN to evolve credible variants of attack evolutions that simulate possible threat mutations in 1.5 h, thus effectively doubling the size of the threat space without introducing noise.

Three examples of real attacks are used to build six additional artificial example threats to provide the basis for a few-shot evolutionary task that alters the configuration settings of detectors in one gradient step set. The increased confidence in detecting a threat increases from 0.54 to 0.87; however, structurally similar benign flows do not exceed a confidence level of 0.25. Thus, false positives are significantly reduced. During this time frame, AUCL continues to aggressively perturb latent space representations to continue to test the confidence of the detector's ability to detect the attack. This results in discovering a significant amount of “epistemic doubt” over 12 h prior to standard detection methods. This increase in uncertainty triggers early escalation and provides a 13-h warning period.

Finally, CETGS develops an evolutionary causal graph of threats (ECGT) based on causal relationships among latent states. The ECGT uses mutation consistency as an edge weight metric and incorporates a calibration component that utilizes predictive accuracy to assign a risk score to each node in the graph based on its state at any given point in time. When the cumulative score of nodes in the graph exceeds 0.7, a zero-day alert is issued along with an interpretable path describing the route the attack took through the system from reconnaissance phase to coordinated data exfiltration. Analysts can use this information to quickly identify where the initial compromise occurred and take corrective action to contain the spread of malware or other forms of cyberattacks in less than 30 min. Furthermore, because the causal graph was constructed based on temporal relationships between latent states, investigators no longer must spend up to 45 min investigating every potential pathway. Evolutionary awareness in the representation space, limited adversarial augmentation, rapid meta-adaptation, and uncertainty calibrated causal reasoning enable more timely, more accurate, and more explainable detection of zero-day threats during operational environments.

### 5.2 Limitations

The paradigm that was suggested functions effectively; however, it does have a few issues. The multi-stage pipeline causes the computer to perform additional work, particularly during training, when AE-GAN synthesis, LMSE encoding, and meta-learning updates need to be improved. When it comes to near-real-time operations, inference latency is sufficient, but, when it comes to large-scale high-throughput applications, it may be necessary to optimize or speed up the application with hardware. Furthermore, the evolutionary drift estimate of AE-GAN makes use of attack data that are arranged in chronological order. This means that samples that are insufficient or do not have accurate time stamps may result in a decrease in the accuracy of the process. Third, cause graphs make CETGS simpler to comprehend; nevertheless, they can quickly become lengthy in crowded areas; therefore, it is necessary to either prune them or summarize them to ensure that analysts can continue to make use of the process. Finally, the system is capable of functioning well with a wide variety of datasets, but its success is still being proven in very particular domains, such as encrypted communications with few apparent features or low-level firmware attacks. These are examples of areas where it is currently being tested.

### 5.3 Recommendations/future scope

The proposed M3-GAZE framework provides interesting research and extension opportunities. Online and continuous learning that incrementally update latent morphology space and evolutionary drift fields without retraining improves long-term idea drift responsiveness. Enterprise network evolutionary knowledge informs detection in developing domains such as automobile or industrial IoT systems using cross-domain transfer learning. CETGS modeling with probabilistic causal inference and counterfactual reasoning should assist analysts predict attack trajectories and evaluate defensive actions before deployments. Moving the platform to federated or privacy-preserving settings will allow organizations to share threat intelligence without releasing personal data samples. Finally, tighter coupling between uncertainty estimations and automated response policies could allow closed-loop defensive systems to dynamically adjust containment strategies based on calibrated risk, enabling fully autonomous, evolution-aware cyber defenses.

## 6 Conclusion

The results of this research demonstrated M3-GAZE, a novel type of threat detection system that identifies zero-day and polymorphic assaults by employing evolutionary inference rather than static classifications. The results of this modification to the design are presented in Tables 2–10, which demonstrate that the performance of the cybersecurity dataset is significantly and reliably improved.

An approach that we proposed improved approach (Sable et al., 2025; Mohamed et al., 2025) and Method (Almheiri et al., 2025) by 10%−20% points was something that we proposed. When applied to CICIDS2017, it achieved zero-day recognition accuracies of up to 94.8%, and it achieved recall levels that were greater than 88% across all datasets. During the strongest baseline, the rate of false negatives for attack families that were not seen reduced from 13% to 6.1%. This represents a significant decrease. This is a significant obstacle for tracking threats in real time. When only five samples are used, researchers have discovered that M3-GAZE has an accuracy rate of more than 86%. The results of this demonstrate that meta-adaptive learning is effective even when there is a limited amount of data. A calibration error value of 0.07 was anticipated for the framework, and the area under the curve (AUC) deterioration during distribution shift was less than 5%.

The framework was stable and strong beyond detection. The early warning times of 14 h and the reduction of the amount of time that analysts had to analyze communications by 35% were both beneficial. These findings demonstrate that the combination of latent morphology modeling, evolution-consistent adversarial enhancement, meta-learning, uncertainty calibration, and causal graph synthesis can result in the creation of zero-day threat detection system sets that are well-balanced and ready to be utilized.

The M3-GAZE framework that was proposed demonstrates significant enhancements in the precision of zero-day detection, recall, calibration, and the capacity to adapt across a variety of cybersecurity datasets. By combining techniques such as adversarial augmentation, meta-adaptive inference, morphology-aware representation learning, and causal reasoning, a solid foundation is established for the subsequent wave of intelligent threat identification. It has been demonstrated that the combination of intelligent Internet of Things (IoT) systems and hybrid deep learning models is particularly effective in making predictions in a variety of domains, including medical diagnostics. It has been demonstrated through this validation across domains that hybrid evolutionary learning systems are capable of being utilized in a wide variety of real-time intelligent monitoring situations (Kavitha and Shaik, 2024) in process.

## Data Availability

Publicly available datasets were analyzed in this study. This data can be found here: https://www.unb.ca/cic/datasets/ids-2017.html.
